# Cyclic di-AMP Oversight of Counter-Ion Osmolyte Pools Impacts Intrinsic Cefuroxime Resistance in Lactococcus lactis

**DOI:** 10.1128/mBio.00324-21

**Published:** 2021-04-08

**Authors:** Huong Thi Pham, Wen Shi, Yuwei Xiang, Su Yi Foo, Manuel R. Plan, Pascal Courtin, Marie-Pierre Chapot-Chartier, Eddy J. Smid, Zhao-Xun Liang, Esteban Marcellin, Mark S. Turner

**Affiliations:** a School of Agriculture and Food Sciences, University of Queensland, Brisbane, Queensland, Australia; b The University of Danang, University of Science and Technology, Da Nang, Vietnam; c Australian Institute for Bioengineering and Nanotechnology, University of Queensland, Brisbane, Queensland, Australia; d Metabolomics Australia, AIBN, The University of Queensland, Brisbane, Queensland, Australia; e Université Paris-Saclay, INRAE, AgroParisTech, Micalis Institute, Jouy-en-Josas, France; f Laboratory of Food Microbiology, Wageningen University and Research, Wageningen, The Netherlands; g School of Biological Sciences, Nanyang Technological University, Singapore; h Queensland Alliance for Agriculture and Food Innovation, University of Queensland, Brisbane, Queensland, Australia; University of California, Davis; Nanyang Technological University

**Keywords:** cyclic di-AMP, antibiotic resistance, osmolyte, regulation, *Lactococcus*, lactic acid bacteria, osmolytes, osmoregulation, second messenger

## Abstract

The broadly conserved cyclic di-AMP (c-di-AMP) is a conditionally essential bacterial second messenger. The pool of c-di-AMP is fine-tuned through diadenylate cyclase and phosphodiesterase activities, and direct binding of c-di-AMP to proteins and riboswitches allows the regulation of a broad spectrum of cellular processes. c-di-AMP has a significant impact on intrinsic β-lactam antibiotic resistance in Gram-positive bacteria; however, the reason for this is currently unclear. In this work, genetic studies revealed that suppressor mutations that decrease the activity of the potassium (K^+^) importer KupB or the glutamine importer GlnPQ restore cefuroxime (CEF) resistance in diadenylate cyclase (*cdaA*) mutants of Lactococcus lactis. Metabolite analyses showed that glutamine is imported by GlnPQ and then rapidly converted to glutamate, and GlnPQ mutations or c-di-AMP negatively affects the pools of the most abundant free amino acids (glutamate and aspartate) during growth. In a high-c-di-AMP mutant, GlnPQ activity could be increased by raising the internal K^+^ level through the overexpression of a c-di-AMP-insensitive KupB variant. These results demonstrate that c-di-AMP reduces GlnPQ activity and, therefore, the level of the major free anions in L. lactis through its inhibition of K^+^ import. Excessive ion accumulation in *cdaA* mutants results in greater spontaneous cell lysis under hypotonic conditions, while CEF-resistant suppressors exhibit reduced cell lysis and lower osmoresistance. This work demonstrates that the overaccumulation of major counter-ion osmolyte pools in c-di-AMP-defective mutants of L. lactis causes cefuroxime sensitivity.

## Abstract (precis)

The bacterial second messenger cyclic di-AMP (c-di-AMP) is a global regulator of potassium homeostasis and compatible solute uptake in many Gram-positive bacteria, making it essential for osmoregulation. The role that c-di-AMP plays in β-lactam resistance, however, is unclear despite being first identified a decade ago.

## INTRODUCTION

Cyclic di-AMP (c-di-AMP) is a class of diffusible nucleotide second messengers that transmit signals based on their intracellular concentration (1). Once a c-di-AMP concentration threshold is reached, it is able to bind to protein and riboswitch receptors to alter their activity (2). Most *Firmicutes* contain one diadenylate cyclase (DAC) (named either CdaA or DacA) and one or two phosphodiesterases (PDEs) (GdpP or PgpH), which fine-tune the intracellular c-di-AMP level (3). Despite a significant number of different c-di-AMP receptors being identified, most play a role in the regulation of K^+^ or compatible solute (e.g., carnitine and glycine-betaine) accumulation (2). In several bacterial genera, a common role for c-di-AMP in adaptation to environmental osmolarity has been identified and aligns well with the receptors to which c-di-AMP binds (4). PDE mutants with elevated c-di-AMP grow poorly in high-osmolarity media (5). In contrast, a lack of c-di-AMP can restrict bacterial growth and result in lysis of cells in rich media due to the overaccumulation of a range of osmolytes, including peptides, K^+^, and glycine-betaine (6–10). Growth of *cdaA* mutants can be rescued in high-osmolarity growth media (6, 11) or through the omission of major osmolytes that are imported by bacteria present in growth media (6–8), suggesting that the internal osmotic pressure is high in *cdaA* mutants.

Another common phenotype observed in bacterial mutants with altered c-di-AMP levels is varying resistance to cell wall-acting antibiotics of the β-lactam family. β-Lactam antibiotics inhibit the transpeptidase activity of penicillin-binding proteins needed to cross-link the glycan strands in peptidoglycan. β-Lactam treatment results in decreased peptidoglycan cross-linking and cell lysis (12). In different species, *gdpP* mutants have shown elevated β-lactam resistance (13–15), and *cdaA* (*dacA*) mutants exhibited β-lactam sensitivity (6, 16, 17). Clinical isolates of Staphylococcus aureus that have developed intrinsic β-lactam resistance have also been found to contain destructive mutations in the *gdpP* gene (18–21). The mechanism by which c-di-AMP regulates β-lactam resistance is, however, unclear despite the first evidence of a connection being reported a decade ago. Two hypotheses have been proposed to explain how c-di-AMP might regulate β-lactam resistance. The first is that c-di-AMP regulates cell wall peptidoglycan synthesis. This could be possible in part via the phosphoglucosamine mutase enzyme GlmM, which is involved in peptidoglycan precursor biosynthesis. The *glmM* gene is colocated in an operon with the most common c-di-AMP synthesis gene, and GlmM regulates c-di-AMP synthesis through direct binding to CdaA/DacA (22–24). The second hypothesis is that a lack of c-di-AMP results in the overaccumulation of intracellular osmolytes that increase internal osmotic pressure to such an extent that the β-lactam-weakened cell wall is unable to prevent lysis (5, 6). This is supported by results showing that β-lactam resistance in c-di-AMP-depleted mutants can be rescued in high-osmolarity media containing sucrose or NaCl (6, 14).

In this study, we identified and characterized genes that contribute to sensitivity to the β-lactam antibiotic cefuroxime (CEF) in c-di-AMP synthesis mutants of the model lactic acid bacterium Lactococcus lactis. CEF was chosen since the depletion of c-di-AMP in Bacillus subtilis and Listeria monocytogenes results in strong CEF hypersensitivity (6, 14, 16). We found that the overaccumulation of K^+^ and the major anionic amino acids (Glu and aspartate [Asp]) contributes to CEF sensitivity in L. lactis *cdaA* mutants. CEF-resistant suppressor mutants became sensitive to osmotic stress and lysed significantly less in a hypotonic environment, suggesting that mutants with reduced osmotic pressure have greater stability. We also identified the mechanism by which c-di-AMP regulates the dominant anionic amino acid levels in L. lactis.

## RESULTS

### CEF-resistant suppressors of *cdaA* mutants commonly contain mutations in a K^+^ or amino acid uptake system.

**FIG 1:**
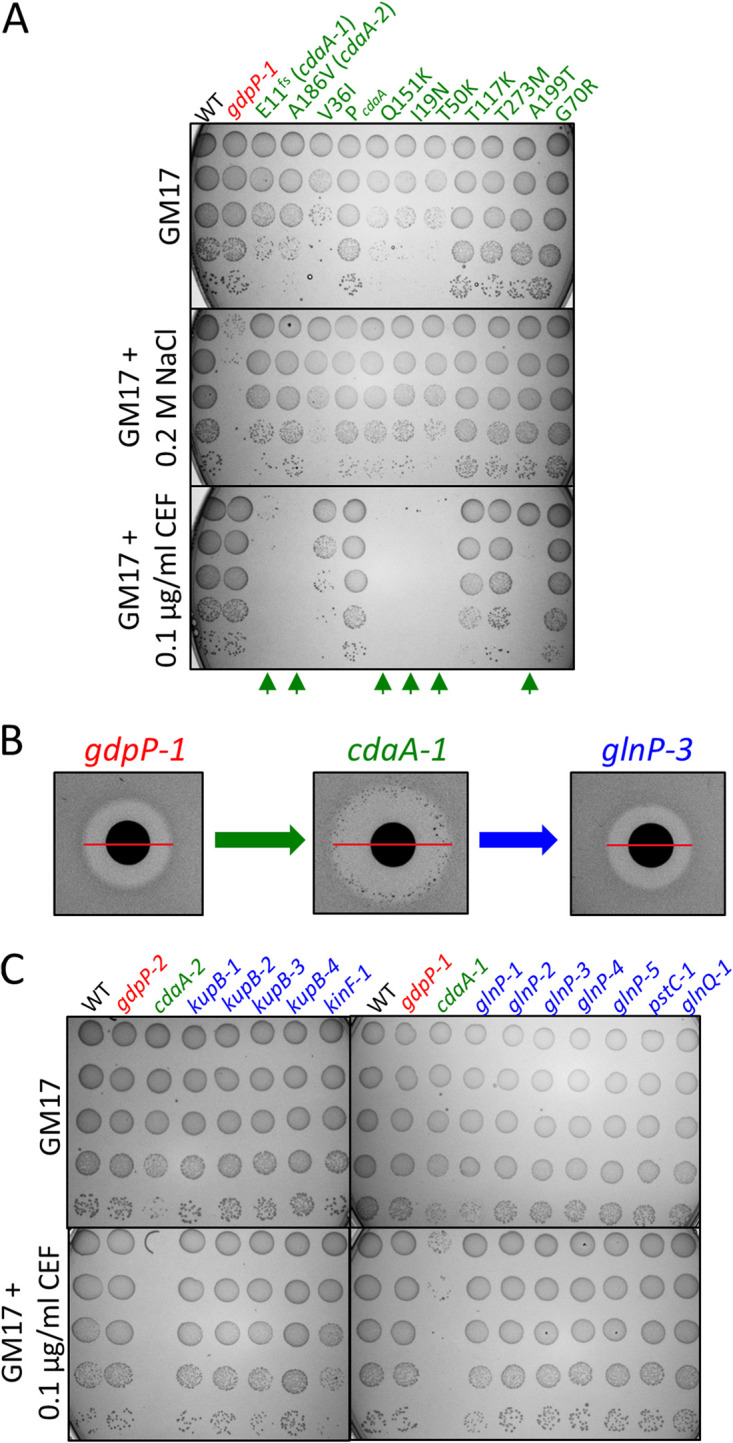
Isolation of cefuroxime-resistant suppressors from mutants defective in *cdaA*. (A) Osmoresistance and CEF resistance of the wild type, a *gdpP* mutant (*gdpP-1*), and a variety of osmoresistant *cdaA* suppressor mutants (labeled with green writing) obtained from parent *gdpP-1* and *gdpP-2* strains. For this and all other dilution spot plates below, spots are 10 μl of 10-fold serial dilutions of mid-log-phase cultures starting from a 10^−1^ dilution at the top. (B) CEF resistance of strains using a disk diffusion assay with 0.15 μg CEF per disk. (C) Confirmation of CEF-resistant suppressors (labeled with blue writing) obtained from the *cdaA-1* and *cdaA-2* strains.

Previously, we identified a large number of independent mutations in *cdaA* in a suppressor screen of different high-c-di-AMP *gdpP* mutants under elevated NaCl concentrations (22, 25). Most of the *cdaA* mutants, which were all more osmoresistant than their *gdpP* mutant parent, contained single amino acid changes in CdaA, while some possessed frameshift mutations or mutations affecting the *cdaA* ribosome-binding site (Fig. 1A). Here, we tested a range of different *cdaA* suppressor mutants for their CEF resistance. Out of 11 *cdaA* mutants, 6 were CEF sensitive (Fig. 1A). We found that in a disk diffusion assay with CEF, strongly growing colonies frequently formed within the inhibition zone of the *cdaA-1* (*cdaA* mutant 1) strain but not its *gdpP-1* parent strain (Fig. 1B). Purification of these colonies and retesting using the disk diffusion assay showed that they underwent one or more mutations to become resistant to CEF (see an example of the *glnP-3* strain in Fig. 1B). To better understand why L. lactis *cdaA* mutant strains are sensitive to CEF, we characterized suppressor mutations that restored CEF resistance.

CEF-sensitive *cdaA-1* and *cdaA-2* strains were chosen for further study. They both have lower c-di-AMP levels than a *gdpP* mutant strain (see Fig. S1 in the supplemental material). They were plated with inhibitory levels of CEF, and 12 suppressor mutants were obtained and confirmed (Fig. 1C). Whole-genome sequencing (WGS) of these suppressors revealed that distinct mutations occurred in suppressors from the two different parent strains *cdaA-1* and *cdaA-2* (Table S1).

10.1128/mBio.00324-21.1 FIG S1 Relative c-di-AMP levels in strains grown in GM17 medium compared to the WT. Data are means ± SD from biological triplicate experiments. Download FIG S1, DOCX file, 0.02 MB. Copyright © 2021 Pham et al. 2021 Pham et al. https://creativecommons.org/licenses/by/4.0/ This content is distributed under the terms of the Creative Commons Attribution 4.0 International license.

10.1128/mBio.00324-21.8 TABLE S1 Mutations identified in CEF suppressor mutants. Download Table S1, DOCX file, 0.01 MB. Copyright © 2021 Pham et al. 2021 Pham et al. https://creativecommons.org/licenses/by/4.0/ This content is distributed under the terms of the Creative Commons Attribution 4.0 International license.

**FIG 2:**
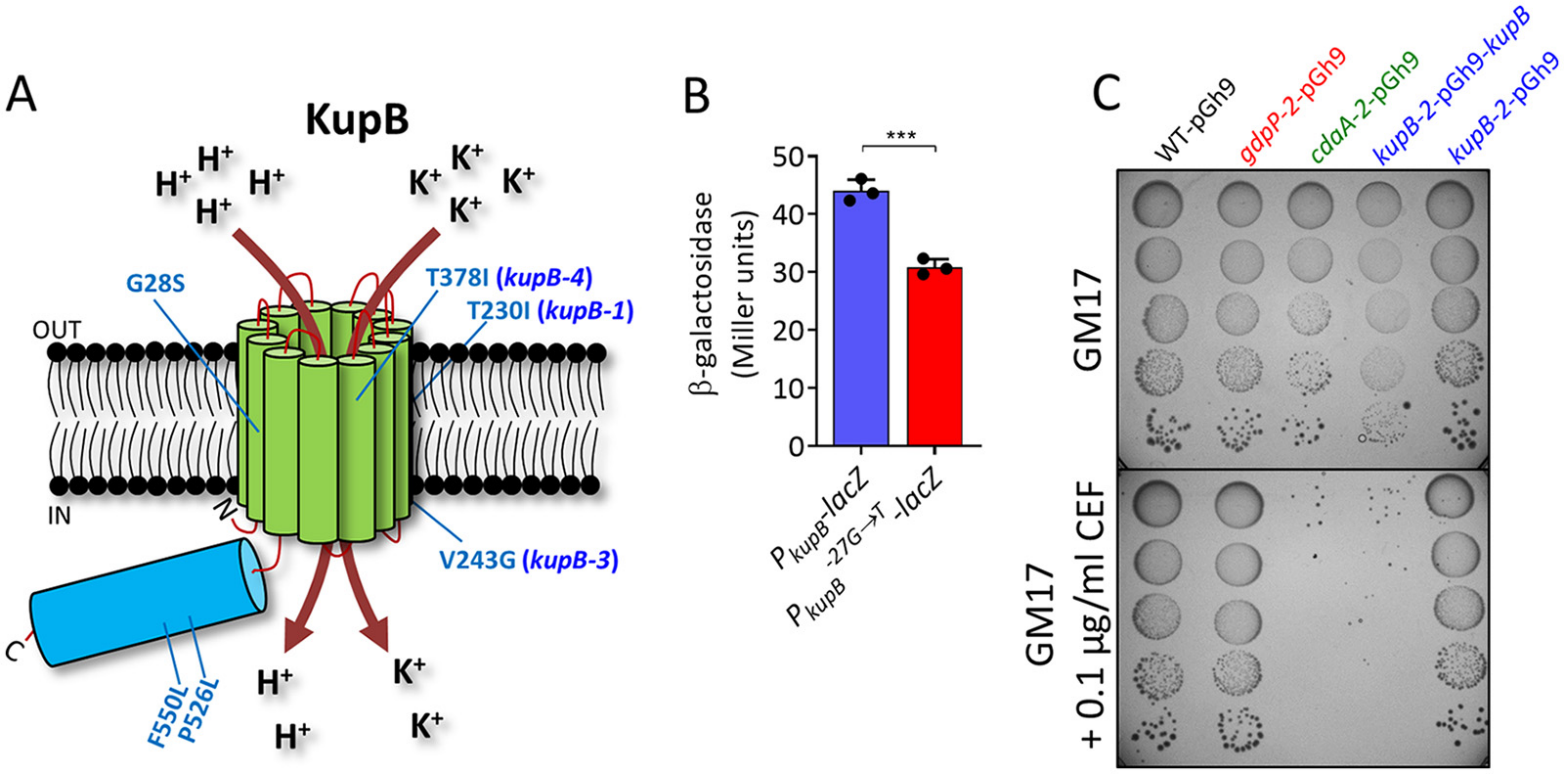
Loss-of-function mutations in *kupB* restore CEF resistance in the *cdaA-2* strain. (A) Location of mutations in KupB in CEF-resistant suppressors of the *cdaA-2* strain. (B) Promoter activity of wild-type and *kupB-2* mutant promoters in the WT strain background. Means ± standard deviations (SD) from three independent biological replicate experiments are shown. ***, *P < *0.001 using an unpaired *t* test. (C) CEF resistance of strains, including the *kupB-2* strain containing a WT copy of *kupB* introduced on the pGh9 plasmid.

For the *cdaA-2* strain, 4 out of 5 CEF-resistant suppressors possessed mutations in *kupB* (Table S1). Additional isolation of CEF-resistant suppressors and sequencing of *kupB* revealed three more independent mutations in this gene (G28S, P526L, and F550L) (Fig. 2A). KupB is a K^+^ importer and has been previously identified as a c-di-AMP receptor protein in L. lactis (26), and gain-of-function mutations in *kupB* restored osmoresistance in a high-c-di-AMP *gdpP* mutant (25). Interestingly, one suppressor strain (*kupB-4*) also contained a transposon insertion upstream of *busAA* (Table S1). Analysis of this IS*905* insertion revealed that it is not oriented in a direction that would provide activation of downstream genes, like that described previously (27, 28). Therefore, the insertion likely disrupts transcription initiated from the native *busAA* promoter located 135 bp upstream.

Interestingly, the KupB amino acid changes identified in our CEF-resistant suppressor screen are mostly located in transmembrane helices within the proximity of residues that interact with K^+^ or protons in KimA from B. subtilis (29) (Fig. S2). To determine if the mutations in *kupB* caused a gain or loss of function, we examined the effect of the mutation 27 bp upstream of *kupB* found in the *kupB-2* strain on gene expression using a *lacZ* reporter. It was found that the G→T mutation reduced expression from the *kupB* promoter by 30% (Fig. 2B). Inspection of the upstream region did not reveal any obvious changes in −10 or −35 sigma factor recognition boxes, so the reason for this downregulation is unclear at this stage. Next, we introduced a wild-type (WT) copy of *kupB* into the *kupB-2* suppressor, which restored CEF sensitivity (Fig. 2C). Taken together, these results demonstrate that reduced K^+^ uptake in the *kupB* suppressor mutants increases CEF resistance.

10.1128/mBio.00324-21.2 FIG S2 Model-based sequence alignment of KimA and Kup homologs and location of the suppressor mutations using the Promals 3d server. Stars indicate mutations identified in CEF-resistant suppressors in KupB. Circles indicate residues identified as being potentially important for K^+^ or proton binding. Jalview was used to prepare the image. Download FIG S2, DOCX file, 0.8 MB. Copyright © 2021 Pham et al. 2021 Pham et al. https://creativecommons.org/licenses/by/4.0/ This content is distributed under the terms of the Creative Commons Attribution 4.0 International license.

**FIG 3:**
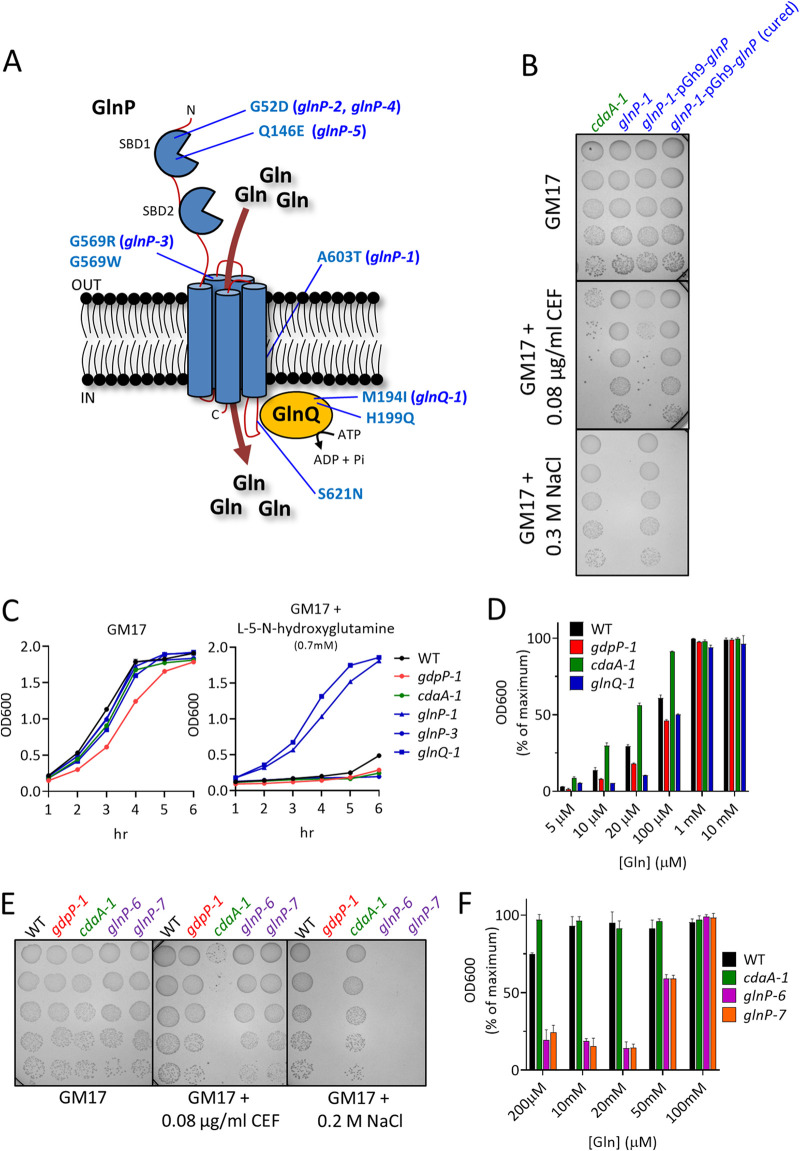
Loss-of-function mutations in GlnPQ restore CEF resistance in the *cdaA-1* strain. (A) Location of GlnPQ mutations in CEF-resistant suppressors obtained from the *cdaA-1* strain. Membrane-spanning regions were predicted using TOPCONS. Note that GlnPQ functions as a homodimer but is shown here as a monomer. (B) CEF and NaCl resistance of strains, including the *glnP-1* strain containing a WT copy of *glnP* introduced on the pGh9 plasmid. A strain in which the plasmid was cured from the *glnP-1*-pGh9-*glnP* strain was also included. (C) Growth of strains with the toxic glutamine analog l-5-*N*-hydroxyglutamine. (D) Growth (after 24 h) of strains in chemically defined medium with various glutamine concentrations. (E) CEF and NaCl resistance of strains, including the l-5-*N*-hydroxyglutamine-resistant suppressors obtained from the *cdaA-1* strain (*glnP-6* and *glnP-7*). (F) Growth (after 24 h) of strains in chemically defined medium with various glutamine concentrations. In panels C, D, and F, means ± SD from three independent biological replicate experiments are shown.

For the *cdaA-1* strain, 6 out of 7 CEF-resistant suppressors contained mutations in the amino acid ATP-binding cassette (ABC) transport system GlnPQ (Table S1). Additional isolation of CEF-resistant suppressors and sequencing of *glnPQ* revealed three more independent mutations in these genes (G569W and S621N in GlnP and H199Q in GlnQ) (Fig. 3A). GlnP is composed of a fusion of two substrate-binding domains (SBDs) to the transmembrane permease domain, and GlnQ is an ATPase (30–33). The primary substrate of GlnPQ is glutamine (Gln); however, other amino acids can be imported through this transporter with various affinities. To determine if the mutations in *glnPQ* caused a gain or loss of function, we introduced the wild-type *glnP* gene in the *glnP-1* strain, which lowered CEF resistance (Fig. 3B). Curing of the *glnP* expression plasmid from this strain resulted in the restoration of CEF resistance (Fig. 3B). Next, we compared the resistances of strains to the toxic Gln analog l-5-*N*-hydroxyglutamine. It was found that 2 of the 3 *glnPQ* suppressor mutants (*glnP-1* and *glnQ-1*) grew well in the presence of the toxic analog (Fig. 3C). It is likely that the *glnP-1* and *glnQ-1* strains contain more destructive *glnPQ* mutations than the *glnP-3* strain, which grew poorly at this concentration of analog tested. Next, we compared the growths of strains in chemically defined medium (CDM) with various Gln levels. The *glnQ-1* strain grew poorly compared with its *cdaA-1* parent strain in low-Gln medium (Fig. 3D). Interestingly, the *cdaA-1* and *gdpP-1* strains grew better and worse, respectively, than the WT at low Gln concentrations (Fig. 3D), suggesting that c-di-AMP may negatively influence the Gln uptake ability.

To obtain a c-di-AMP synthesis-defective strain completely defective in GlnPQ activity, we plated the *cdaA-1* strain onto agar containing an inhibitory concentration of the toxic analog l-5-*N*-hydroxyglutamine. Two analog-resistant suppressors were obtained (Fig. S3), and analysis of *glnP* revealed single nucleotide changes that introduced a TAA stop codon at codon 44 (*glnP-7*) or codon 442 (*glnP-6*). Both the *glnP-6* and *glnP-7* strains were more CEF resistant than their parent *cdaA-1* strain (Fig. 3E). They were also unable to fully grow in CDM unless very high levels of Gln were provided (Fig. 3F), showing that the Gln acquisition ability of the *glnP-6* and *glnP-7* strains is severely impaired. Taken together, these results demonstrate that destructive *glnPQ* mutations lead to reduced uptake of Gln (and possibly other amino acids), which results in CEF resistance in a c-di-AMP synthase-defective strain.

10.1128/mBio.00324-21.3 FIG S3 Confirmation of l-5-hydroxyglutamine resistance of the suppressors *glnP-6* and *glnP-7* that were derived from the *cdaA-1* strain. Download FIG S3, DOCX file, 1.7 MB. Copyright © 2021 Pham et al. 2021 Pham et al. https://creativecommons.org/licenses/by/4.0/ This content is distributed under the terms of the Creative Commons Attribution 4.0 International license.

### CEF-resistant suppressors possess an osmosensitive phenotype.

We hypothesized that the CEF-resistant suppressors have lower concentrations of intracellular osmolytes (K^+^ or free amino acid pool), which either directly or indirectly results in reduced internal osmotic pressure leading to greater cell stability during CEF-induced cell wall weakening. A lower level of intracellular osmolytes would also be expected to reduce osmoresistance. The CEF-resistant suppressors tested were all found to be more sensitive to osmotic stress than their parent strains (*cdaA-1* or *cdaA-2*) (Fig. S4A). Some CEF-resistant strains (*kupB-1*, *kupB-4*, *glnP-1*, and *glnQ-1*) were found to be highly NaCl sensitive, suggesting that their mutations are more severe. The expression of *glnP* rescued NaCl resistance in the *glnP-1* strain (Fig. 3B), and the l-5-*N*-hydroxyglutamine-selected suppressors *glnP-6* and *glnP-7* were highly NaCl sensitive (Fig. 3E), confirming that in *cdaA* mutants, GlnPQ activity is required for growth under high osmolarity. We next determined if higher growth medium osmolarity could rescue the CEF resistance of the *cdaA-1* and *cdaA-2* strains. It was found that the addition of increased NaCl enhanced the growth of these strains on CEF-containing agar (Fig. S4B). Together, these data show that mutations that allow for CEF resistance lower the osmotic pressure within the cell, and CEF-sensitive *cdaA* mutant cells can be stabilized by elevated external osmolarity.

10.1128/mBio.00324-21.4 FIG S4 (A) CEF-resistant suppressors (labeled in blue) have reduced osmoresistance compared to their CEF-sensitive *cdaA-1* and *cdaA-2* parent strains (labeled in green). Strains were grown on GM17 agar with various levels of additional NaCl. (B) NaCl improves CEF resistance in *cdaA-1* and *cdaA-2* strains. Strains were grown on GM17 agar with or without CEF and various levels of additional NaCl. Download FIG S4, DOCX file, 1.5 MB. Copyright © 2021 Pham et al. 2021 Pham et al. https://creativecommons.org/licenses/by/4.0/ This content is distributed under the terms of the Creative Commons Attribution 4.0 International license.

### CEF-resistant suppressors exhibit reduced cell lysis.

**FIG 4:**
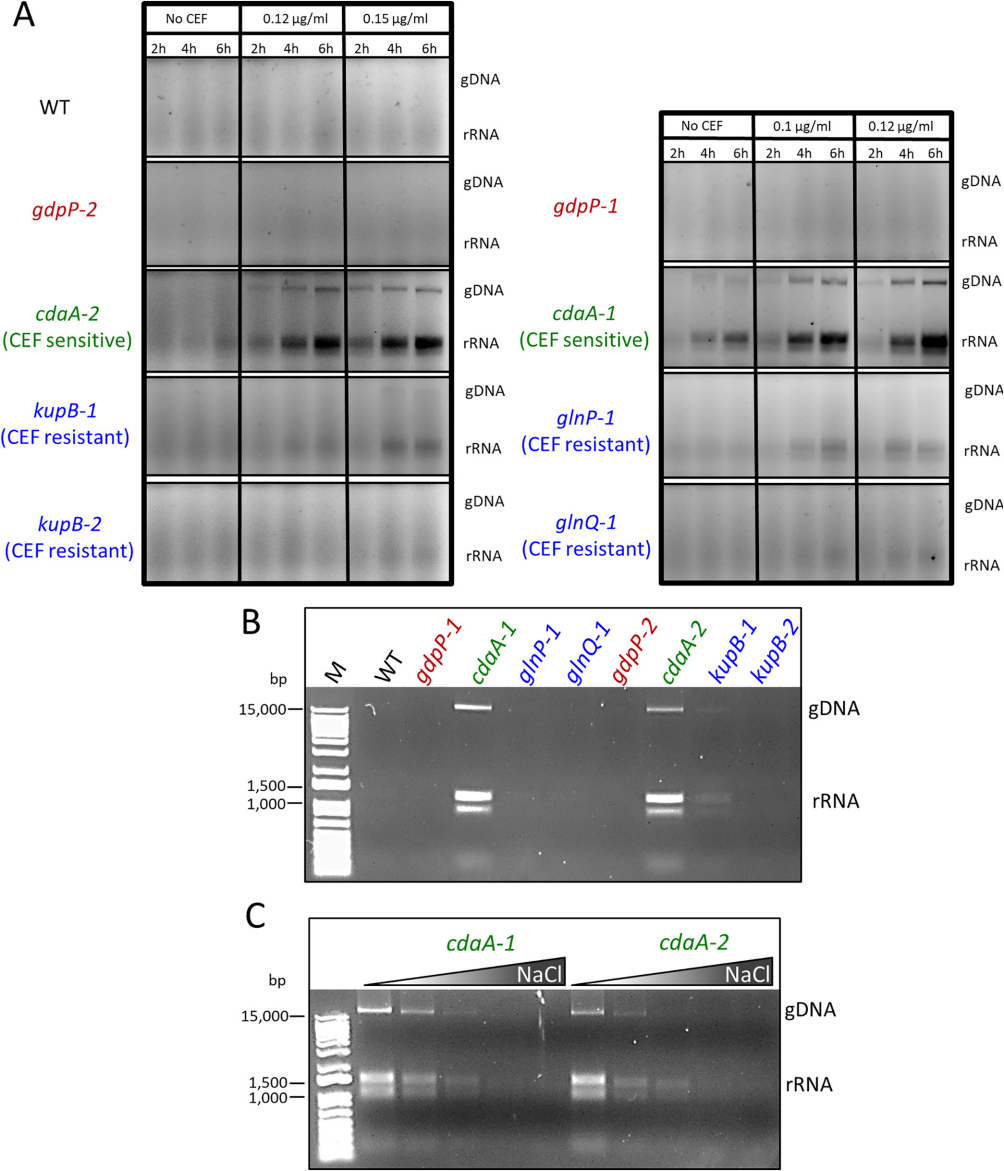
CEF-resistant suppressors release less DNA and RNA during growth with CEF or following resuspension in water. (A) Agarose gel electrophoresis of supernatants from strains grown for various times in HI broth with various CEF concentrations. The locations of genomic DNA (gDNA) and rRNA are indicated. (B) Agarose gel electrophoresis analysis of the supernatant following resuspension of cells (grown to mid-log phase in GY medium) in water. (C) Agarose gel electrophoresis analysis of the supernatant following resuspension of cells (grown to mid-log phase in GY medium with different NaCl concentrations) in water. The gradient from left to right for each strain indicates cells grown in GY medium with 0, 0.05, 0.1, 0.15, and 0.2 M NaCl. The M lane indicates the DNA ladder.

In c-di-AMP-depleted mutants of B. subtilis and L. monocytogenes, elevated cell lysis occurs during growth in rich media (14, 16). We examined if *cdaA* mutants of L. lactis would exhibit greater lysis during growth with CEF. Culture supernatants were examined for the presence of DNA and RNA by gel electrophoresis as an indicator of cell lysis. Both the *cdaA-1* and *cdaA-2* strains were found to lyse significantly when cultured with CEF, while CEF-resistant suppressors, the WT, and *gdpP* mutants showed no or minimal lysis (Fig. 4A). L. lactis *cdaA-1* was also found to undergo some lysis during growth in medium without CEF (Fig. 4A). To explore the role of osmotic pressure in the stability of strains, we compared the amounts of spontaneous lysis of washed cells grown to mid-log-phase, which were suspended in pure water (a hypotonic solution). L. lactis *cdaA-1* cells grown in GM17 and heart infusion (HI) media were relatively stable when suspended in water; however, cells grown in glucose-yeast (GY) broth were much more prone to lysis. It was found that both the *cdaA-1* and *cdaA-2* strains lysed more in water than CEF resistance suppressors, the WT, and *gdpP* mutants (Fig. 4B). To lower the internal osmotic pressure of cells, *cdaA* mutants were pregrown in GY medium with increasing concentrations of NaCl. This resulted in greater stability of cells following their resuspension in water, most likely due to a lowering of cell turgor pressure (Fig. 4C). These results suggest that c-di-AMP synthesis mutants possess high internal osmotic pressure, which leads to reduced cell stability.

We next examined if there are cell wall peptidoglycan changes in c-di-AMP-defective strains that may be contributing to CEF sensitivity and reduced cell stability. Remarkably, the *cdaA-1* strain, which is CEF sensitive and exhibited less stability than the other strains, had a cell wall that was the same thickness as that of the WT and thicker than the cell walls of the *gdpP-1* and *glnP-1* strains (Fig. S5A). This suggests that a defect in c-di-AMP synthesis in L. lactis does not negatively affect cell wall biosynthesis. In previous work, we found that an L. lactis *gdpP* mutant contained elevated peptidoglycan precursor UDP-*N*-acetylglucosamine (UDP-NAG) levels, which were lowered upon mutation of the phosphoglucosamine mutase gene *glmM* (22). Here, we measured UDP-NAG levels and found that they negatively correlated with cell wall thickness. UDP-NAG levels were higher in mutants with thinner cell walls, indicating that slower cell wall biosynthesis may result in the accumulation of peptidoglycan precursors (Fig. S5B). Peptidoglycan muropeptides and the peptidoglycan cross-linking index of strains were also analyzed (Fig. S5C). Since CEF inhibits peptidoglycan cross-linking (34), cells with reduced cross-linking may exhibit greater sensitivity. However, a small but statistically significant increase in cross-linking was observed in the CEF-sensitive *cdaA-1* mutant compared to its parent *gdpP-1* strain (Fig. S5C). Taken together, cell wall analyses did not provide an explanation for the variations in CEF resistance and cell integrity observed in the strains examined here.

10.1128/mBio.00324-21.5 FIG S5 Cell wall peptidoglycan and UDP-*N*-acetylglucosamine analyses. (A) Cell wall thickness measurements of strains using transmission electron microscopy (TEM), with means ± SD (bars) and the number of measurements taken (*n*) indicated. ***, *P < *0.001 (by one-way ANOVA followed by Tukey’s test for multiple comparisons). (B) UDP-*N*-acetylglucosamine levels in strains. Data (means ± SD) are from three independent biological replicate experiments. **, *P < *0.01; ***, *P < *0.001 (by a two-tailed *t* test). NS, not significant. (C) Percent abundances of muropeptides and peptidoglycan cross-linking indices in different strains. Data (means ± SD) are from three independent biological replicate experiments. *, *P < *0.05; ***, *P < *0.001 (by one-way ANOVA followed by Tukey’s test for multiple comparisons for crosslinking index results). Download FIG S5, DOCX file, 0.04 MB. Copyright © 2021 Pham et al. 2021 Pham et al. https://creativecommons.org/licenses/by/4.0/ This content is distributed under the terms of the Creative Commons Attribution 4.0 International license.

### Mutations in GlnPQ lower intracellular Gln, Glu, and Asp levels.

While the roles of K^+^ and the c-di-AMP-binding receptor KupB have been studied previously in the osmoresistance of L. lactis (25, 26), the role of GlnPQ in c-di-AMP-regulated processes has not been reported. GlnPQ has been found to bind and transport 3 different amino acids (Gln, Glu, and Asn) with different affinities and rates (30, 32, 33). GlnPQ transports only the protonated form of Glu, not the anion, which is the dominant species at physiological pH, and its capacity to transport Asn is lower than its capacity to transport Gln (33). It is the sole transport system in L. lactis for the essential amino acids Gln and Glu (32). This work points to Gln being the primary target of GlnPQ.

**FIG 5:**
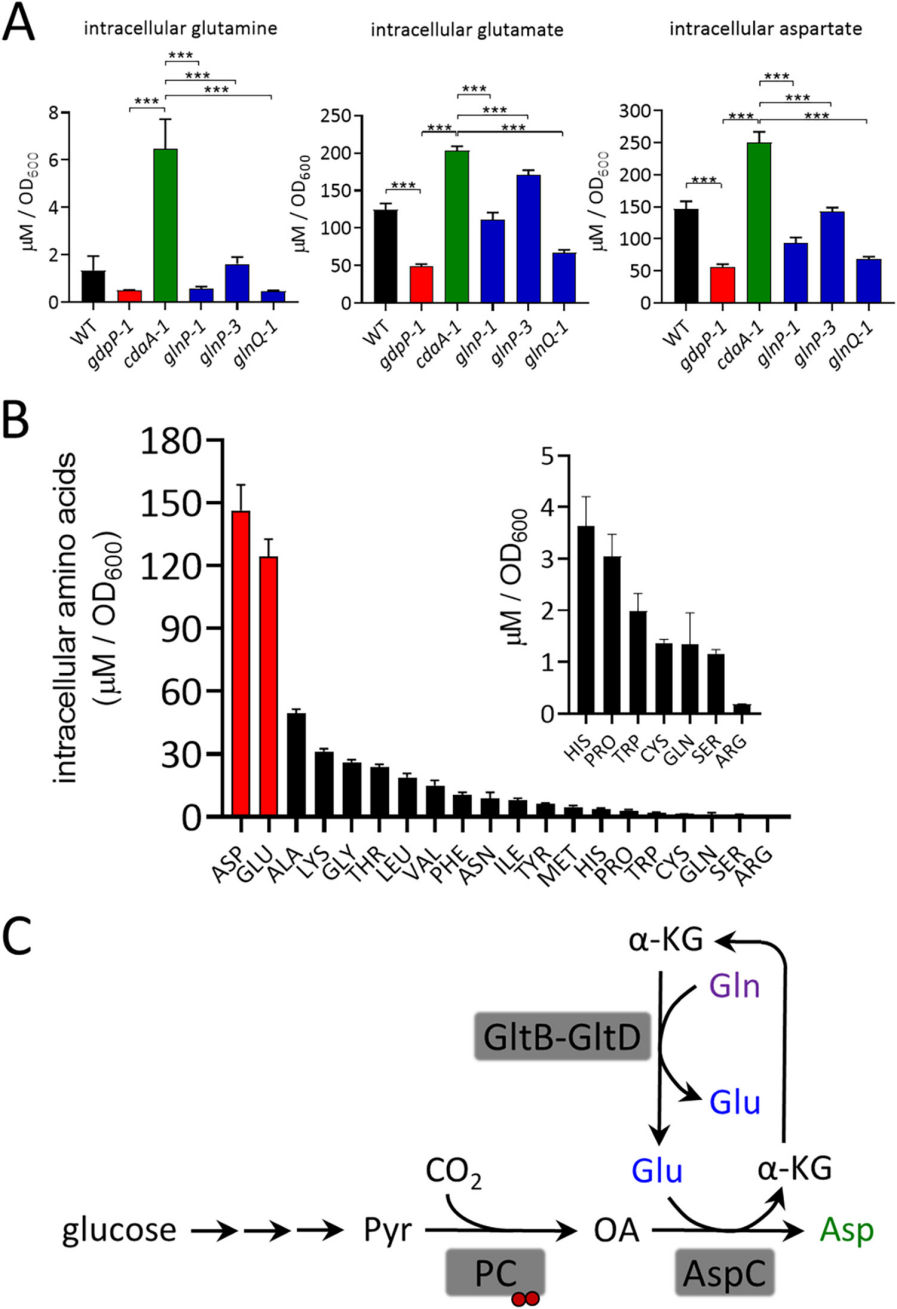
GlnPQ mutations lower the levels of Gln, Glu, and Asp in CEF-resistant suppressors. (A) Intracellular levels of Gln, Glu, and Asp in the indicated strains. Data are means ± SD from three independent biological replicate experiments. ***, *P < *0.001 using one-way analysis of variance (ANOVA) followed by Tukey’s test for multiple comparisons. (B) All intracellular free amino acid levels in WT L. lactis. The inset shows amino acids of low abundance. Data are means ± SD from three independent biological replicate experiments. (C) Proposed pathway for the conversion of Gln to Glu and Asp in L. lactis. Note that several other enzymes in L. lactis can convert Gln to Glu during nucleotide, nucleotide-sugar, and amino acid biosynthesis. Pyr, pyruvate; OA, oxaloacetate; α-KG, α-ketoglutarate; GltB and GltD, glutamate synthase large and small subunits, respectively; AspC, aspartate aminotransferase. The red circles depict c-di-AMP binding to pyruvate carboxylase (PC).

The CEF-sensitive *cdaA-1* strain contained a higher level of Gln than its parent *gdpP-1* strain, which had a low level like the *glnPQ* suppressors (Fig. 5A). Quantitation of all free amino acid levels in WT L. lactis revealed that Asp and Glu were by far the most abundant, present at ∼100-fold-higher concentrations than Gln (Fig. 5B). Interestingly, their levels also varied significantly between the strains tested. The *cdaA-1* strain contained significantly higher Glu and Asp levels than its CEF-resistant *glnPQ* suppressors (Fig. 5A). In addition, the c-di-AMP level had a negative effect on Glu and Asp levels, with *cdaA-1* and *gdpP-1* mutants having significantly higher and lower levels, respectively, than their parents (Fig. 5A). From this, we hypothesized that following import by GlnPQ, Gln is converted to Glu and Asp, which together form a major anionic solute pool. A proposed pathway showing the conversion of Gln to Glu and Asp in L. lactis is shown in Fig. 5C. The pathway also includes the c-di-AMP receptor pyruvate carboxylase (PC), which synthesizes the Asp precursor oxaloacetate. From these results, it was of interest to investigate how c-di-AMP levels might also affect GlnPQ activity and conversion of Gln to Glu and Asp.

### Gln uptake by GlnPQ is activated by increased intracellular ionic strength and K^+^, which provides high levels of the counter-ion Glu.

**FIG 6:**
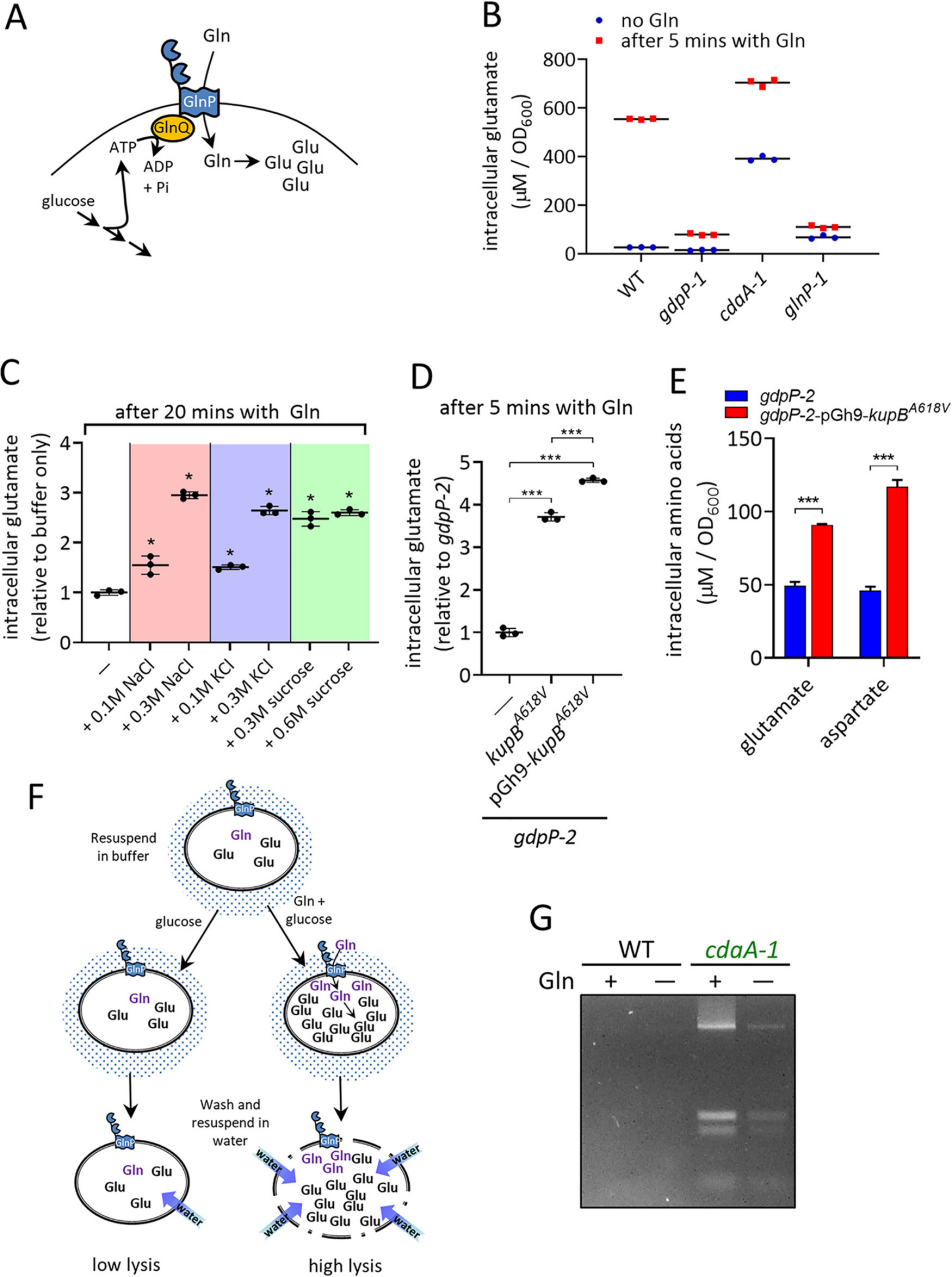
GlnPQ activity controls intracellular Glu in response to intracellular K^+^, and overaccumulation of Glu leads to greater cell instability in the *cdaA-1* strain. (A) Pathway showing ATP-dependent Gln uptake by GlnPQ and conversion to Glu. (B) Intracellular Glu levels in resting cells following 5 min of incubation with glucose with or without Gln. The means from three independent biological replicate experiments are shown as horizontal bars. (C) Effect of increased osmolarity on Gln uptake in resting cells of the WT after 20 min in buffer. Levels are relative to those in cells in buffer with no additional osmolyte added. *, *P < *0.001 using one-way ANOVA followed by Tukey’s test for multiple comparisons. (D) Effect of expression of the c-di-AMP-insensitive KupB variant KupB^A618V^ in a single copy (*gdpP-2–kupB^A618V^*) and multiple copies (*gdpP-2*-pGh9-*kupB^A618V^*) on Gln uptake by resting cells of the high-c-di-AMP *gdpP-2* mutant in buffer. ***, *P < *0.001 using one-way ANOVA followed by Tukey’s test for multiple comparisons. (E) Intracellular Glu and Asp levels in *gdpP-2* cells expressing the c-di-AMP-insensitive K^+^ importer KupB^A618V^ in cells grown in GM17 medium. (F) Model for enhanced lysis in Glu-loaded cells. (G) DNA and RNA release from cells resuspended in water after being grown in CDM with a low glutamine concentration (100 μM) and then incubated for 30 min with glucose with or without Gln in buffer. Quantitative data are presented as means ± SD from three independent biological replicate experiments.

The experiments described above were carried out using cells grown in rich complex media, so intracellular amino acids can derive from both imported peptides as well as free extracellular amino acids. To verify that the changes in Glu and Asp levels seen in strains were specifically due to altered import of Gln by GlnPQ, we carried out Gln feeding assays with resting (nongrowing) cells in buffer with glucose (Fig. 6A). In the WT, rapid and large increases in intracellular Gln and Glu levels were observed; however, no increase in Asp was observed even after 60 min with Gln (Fig. 6B; Fig. S6). This suggests that Gln/Glu is not used to generate Asp in resting cells. We therefore used intracellular Glu as a marker for GlnPQ activity since its level continued to rise over 60 min, while the level of Gln fell due to its conversion to Glu. This confirms that following ATP-dependent import of Gln, rapid conversion to Glu occurs. The high-c-di-AMP *gdpP* mutant strain *gdpP-1* was unable to increase its Glu level to high levels, and its levels remained around 7-fold lower than that of the WT after 5 min (Fig. 6B). The restricted increase in Glu in the *gdpP-1* strain was the result of reduced activity of GlnPQ since intracellular Gln levels measured after 5 min of feeding with Gln were also much lower (∼8-fold) than those of the WT. The *cdaA-1* strain contained high initial levels of Glu but further increased this level following Gln feeding to remain higher than that of the WT. The *glnP* suppressor mutant *glnP-1* had a low starting level of Glu and following Gln addition remained low, as expected, since the GlnPQ transporter is defective in this strain.

10.1128/mBio.00324-21.6 FIG S6 Gln, Glu, and Asp levels change over 60 min after Gln feeding to WT energized resting cells. Data are shown as means ± SD. Download FIG S6, DOCX file, 0.02 MB. Copyright © 2021 Pham et al. 2021 Pham et al. https://creativecommons.org/licenses/by/4.0/ This content is distributed under the terms of the Creative Commons Attribution 4.0 International license.

The finding that the *gdpP* mutant strain *gdpP-1* is unable to strongly increase Glu levels in this assay suggests that GlnPQ activity is inhibited in this strain. The *gdpP-1* strain contains a high level of c-di-AMP (22), which leads to inhibition of K^+^ import through direct binding to KupB (25, 26). We hypothesized that GlnPQ activity is affected by ionic strength in L. lactis cells and that the low K^+^ level observed in the *gdpP* mutant prevents the activation of GlnPQ. Exposing cells to elevated external osmotic conditions, which we predicted would increase the intracellular ionic strength, led to significantly higher intracellular Glu levels (Fig. 6C).

Next, we examined if an increase in intracellular K^+^ in a high-c-di-AMP *gdpP* mutant could activate GlnPQ. The *gdpP-2* strain containing either a single copy or multiple copies of a constitutively active *kupB^A618V^* gene variant accumulated significantly higher Glu levels following Gln feeding (Fig. 6D). Levels of Glu and Asp in the *gdpP-2* strain expressing *kupB^A618V^* were also significantly higher in cells during growth in rich media (Fig. 6E). Therefore, the level of intracellular K^+^, which is inhibited by c-di-AMP binding to KupB, positively affects the accumulation of the major counter-ion amino acids Glu and Asp in L. lactis.

Finally, we were interested in determining if overfeeding of Gln to *cdaA-1* cells would trigger greater cell lysis in nongrowing cells. We grew *cdaA-1* cells in CDM with low Gln levels to decrease the Glu pool and reduce cell lysis. Cells were then incubated with either glucose only or glucose and Gln (Fig. 6F), washed, and then resuspended in water. It was found that *cdaA-1* cells incubated with glucose and Gln lysed more than the same batch of cells incubated with only glucose (Fig. 6G). Since this assay was performed using nongrowing cells, this confirms that lysis is the direct result of Gln uptake (and Glu overaccumulation) and is unrelated to a change in a metabolic process (e.g., cell wall biosynthesis). Gln feeding to the WT did not result in any observable increase in lysis, which suggests that its osmotic pressure is still lower than that in the *cdaA* mutant. This is likely due to the intact c-di-AMP system in the WT lowering the levels of other osmolytes within the cell, unlike the *cdaA* mutant.

## DISCUSSION

c-di-AMP has emerged as a global osmoregulatory signal in numerous bacterial genera (35). Evidence has indicated that the link between c-di-AMP and β-lactam antibiotic resistance is an additional consequence of its regulation of intracellular osmolyte levels (5, 6). This hypothesis proposes that cells possessing higher internal osmotic pressure (i.e., *cdaA* mutants) will be more susceptible to osmotic lysis, especially when the stress-bearing peptidoglycan layer is compromised upon CEF exposure. Here, in a screen for suppressors that rescued the CEF resistance of partially defective *cdaA* L. lactis mutants, mutations that lowered the levels of major inorganic or organic ions (K^+^, Glu, and Asp) were found. GlnPQ and KupB suppressors grew poorly on media with elevated salt, demonstrating that these ions play important roles in osmoregulation in L. lactis. In nongrowing cells, Gln uptake (and Glu accumulation) triggered greater lysis of an L. lactis *cdaA* mutant, providing further evidence for osmotic pressure being an important contributor to cell instability in a strain with defective c-di-AMP synthesis. Reduced ion accumulation has also been observed in CEF-resistant suppressors of an L. monocytogenes *cdaA* mutant (6). Mutations that restricted PC activity led to lower levels of the citrate anion. With respect to a direct role of c-di-AMP in cell wall homeostasis, differences in peptidoglycan cross-linking or precursor synthesis have been identified previously in high-c-di-AMP mutants (13, 22, 36). Our analysis of peptidoglycan thickness, cross-linking, and precursor levels, however, did not reveal alterations that would appear to render the L. lactis *cdaA* mutant peptidoglycan more susceptible to CEF. Indeed, the high-c-di-AMP *gdpP* mutant, which had the thinnest cell wall and the least cross-linked peptidoglycan, was CEF resistant. A role for more subtle cell wall changes in CEF resistance, however, cannot be excluded. Therefore, our findings provide additional support for a model whereby *cdaA* mutants accumulate unhealthy levels of several different osmolytes, which, when combined, lead to a critical internal osmotic pressure that a normally structured cell wall is unable to fully withstand.

In support of this theory, we identified that the *cdaA-1* mutant contains a *busAA-AB* promoter mutation that destroys the transcription of the transporter for the compatible solute glycine-betaine (see Table S2 and Fig. S7 in the supplemental material). This mutation likely permitted the growth of this suppressor, which contains a severe *cdaA* frameshift mutation, on normal media. This is similar to that seen in a Streptococcus agalactiae *cdaA* mutant, which was viable only after the inactivation of its glycine-betaine transporter (8). Although viable, the L. lactis *cdaA-1* mutant is still sensitive to CEF, and a subsequent lowering of additional osmolytes through the inactivation of GlnPQ is necessary to restore CEF resistance. Therefore, c-di-AMP-controlled osmolytes likely have an additive effect on internal osmotic pressure whereby moderate osmolyte overaccumulation permits growth but generates a CEF-sensitive phenotype, while extreme osmolyte overaccumulation results in a complete loss of viability. It is well established that the turgor pressure of Gram-positive bacteria is up to 10-fold higher than that of Gram-negative bacteria and therefore needs to be tightly controlled (37). Our findings and those of others (5–9, 11, 25, 38) suggest that phenotypes affecting the growth and cell integrity of low- and high-c-di-AMP mutants can in most part be explained by variations in the levels of turgor-inducing internal osmolytes.

10.1128/mBio.00324-21.7 FIG S7 The mutation upstream of *busAA* in the *cdaA-1* strain is located in the *busAA* promoter and abolishes promoter activity. (A) Location of the G→T mutation 142 bp upstream of *busAA* in the *cdaA-1* strain. (B) Activities of *lacZ* fusions in pTCV-lac were evaluated in the L. lactis WT. The WT *busAA* promoter (containing a G nucleotide at position 142) and the *cdaA-1 cdaA-1* promoter (containing a T nucleotide at position 142) were compared. Three 10-μl spots of each strain were spotted and grown on GM17 agar with 0.1 M NaCl. Download FIG S7, DOCX file, 2.1 MB. Copyright © 2021 Pham et al. 2021 Pham et al. https://creativecommons.org/licenses/by/4.0/ This content is distributed under the terms of the Creative Commons Attribution 4.0 International license.

10.1128/mBio.00324-21.9 TABLE S2 Strains and plasmids used in this study. Download Table S2, DOCX file, 0.03 MB. Copyright © 2021 Pham et al. 2021 Pham et al. https://creativecommons.org/licenses/by/4.0/ This content is distributed under the terms of the Creative Commons Attribution 4.0 International license.

**FIG 7:**
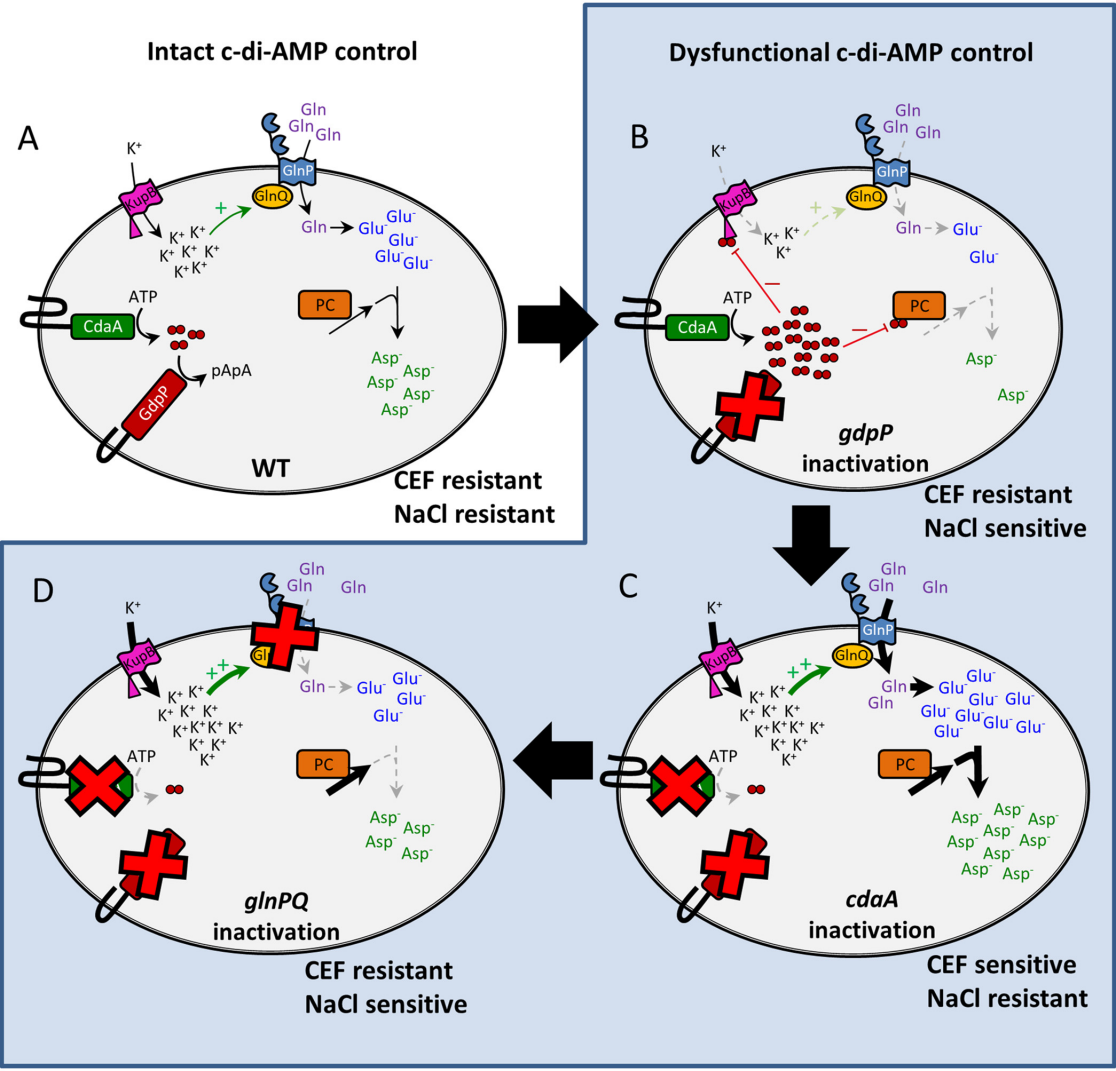
Proposed model for changes leading to CEF sensitivity in the *cdaA-1* strain and CEF resistance in *glnPQ* suppressor mutant strains. (A) The WT normally contains low c-di-AMP levels, resulting in high K^+^ uptake by KupB. High intracellular K^+^ levels lead to active Gln import by GlnPQ and conversion to high levels of anionic Glu and Asp. PC is also uninhibited in this strain and contributes to the Asp pool. WT cells can modulate their c-di-AMP level in response to environmental stressors. (B) Inactivation of GdpP in the *gdpP-1* strain results in a high level of c-di-AMP, which binds and reduces K^+^ uptake by KupB. Lower intracellular K^+^ concentrations result in less activation of GlnPQ and smaller Glu and Asp pools. PC is also inhibited in this strain by c-di-AMP. This strain is highly salt sensitive due to low levels of intracellular osmolytes. (C) Selection for salt-resistant mutants of the *gdpP-1* strain resulted in partial CdaA inactivation and low-level c-di-AMP synthesis, leading to very high K^+^ uptake by an uninhibited KupB. A high level of K^+^ strongly activates Gln uptake by GlnPQ, and high Glu and Asp pools form. In this mutant, PC is also not inhibited by c-di-AMP and increases the production of the Asp precursor oxaloacetate. This mutant is CEF sensitive due to osmolyte overaccumulation. (D) Selection of CEF-resistant suppressors of the *cdaA-1* strain results in partial inactivation of GlnPQ, which lowers Gln uptake and the major pools of Glu and Asp. This reduces major intracellular anions. PC is still uninhibited in this strain, but the lower levels of Glu result in lower levels of Asp. c-di-AMP is depicted as two red circles, and its breakdown product pApA is 5′-phosphadenylyl-adenosine. Black or gray arrows show transport or enzymatic conversion steps. Thick arrows indicate greater flux, while dashed arrows indicate reduced flux. Green arrows indicate activation of GlnPQ by K^+^. Red lines indicate binding and inhibition of KupB and PC by c-di-AMP.

In L. lactis, c-di-AMP negatively regulates Asp, K^+^, and glycine-betaine levels through direct binding to PC, Kup homologs, BusR, and BusAA/OpuAA (25, 26, 39, 40). Its regulatory reach can now be extended to the control of Glu (and Asp) levels through indirect regulation of GlnPQ. Glu is the major anion in most bacteria, and its accumulation allows the cell to balance the charge of significant levels of K^+^ (41, 42). In the L. lactis WT, Glu is present at a high level, but the other anionic amino acid Asp is also present at a similar concentration. Together, these two amino acids represent 55% of the total free amino acids in WT L. lactis (Fig. 5B). In L. lactis, Glu (and Gln) is unable to be synthesized due to an incomplete tricarboxylic acid (TCA) cycle and needs to be sourced from peptides or amino acids external to the cell (33). The GlnPQ transporter in L. lactis plays an important role through its ability to efficiently transport Gln with both high and low affinities (33). Studies of L. lactis growing in defined media revealed that out of 20 amino acids provided to cells in defined media, Gln was the most consumed amino acid, accounting for up to 50% of the total nitrogen imported (43). Therefore, Gln uptake and conversion are significant processes in L. lactis and allow the cell to generate large osmolyte pools of Glu and Asp. Here, we provide evidence that in L. lactis, c-di-AMP has a significant influence on GlnPQ transporter activity through its control of K^+^ levels via direct binding to KupB (25, 26, 44). c-di-AMP can therefore modulate the levels of the major K^+^ counter-ions Glu and Asp. The presence of structurally and functionally similar GlnPQ transport systems in streptococci and enterococci (30) suggests that this regulation mechanism likely extends beyond L. lactis. Based on our work and others, a model for c-di-AMP-regulated homeostasis of the major intracellular ions and their effect on CEF resistance in L. lactis can be proposed (Fig. 7).

Recent work in S. aureus and B. subtilis identified suppressor mutations in Gln and Glu transporters, respectively, which rescued the growth of mutants devoid of c-di-AMP (9, 38). These results align well with those found in L. lactis and suggest that cells defective in c-di-AMP production are unable to regulate intracellular levels of major osmolyte amino acids. The mode of regulation of the mutated transporters (AlsT, AimA, and YfkC) is not known; however, it would be of interest to explore if they are controlled indirectly by c-di-AMP, like GlnPQ in L. lactis. In support of our findings, a previous *in vitro* characterization of L. lactis GlnPQ found that Gln uptake in reconstituted proteoliposomes was activated ∼4-fold by an increased salt concentration in the lumen (32). In other work, the ATPase component (GlnQ) of S. agalactiae was found not to bind c-di-AMP (8), therefore making it unlikely that c-di-AMP-regulated Gln uptake occurs via a direct interaction. Indeed, when we overexpressed constitutively active KupB^A618V^ in the *gdpP-2* strain, which triggers an accumulation of c-di-AMP (25), GlnPQ was found to be more active due to elevated K^+^ import (Fig. 6D). Recent work in B. subtilis has found that Glu availability affects K^+^ import activity by KtrCD (45), further confirming the need for alignment of ionic osmolyte levels. Coordination of K^+^ and anion levels ensures a balancing of the charge within the cell, and by controlling both through a common orchestrator (c-di-AMP), it can occur efficiently and rapidly.

## MATERIALS AND METHODS

### Strains, media, and chemicals used.

L. lactis strains (see Table S2 in the supplemental material) were routinely grown in nonshaking tubes at 30°C in M17 medium (Difco, USA) supplemented with 0.5% (wt/vol) glucose (GM17). When needed, 3 μg/ml erythromycin (Em) was added to the media for L. lactis. DNA release assays were carried out on cells grown in either heart infusion (HI) medium (Oxoid) or glucose-yeast (GY) medium (1.33% yeast extract [Oxoid], 1.33% glucose, and 1% 0.1 M K_2_HPO_4_). To characterize the growth of L. lactis under different concentrations of glutamine (Gln), chemically defined minimal medium (CDM) using two types of Dulbecco’s modified Eagle’s medium (DMEM) (Merck) was used as the base (39). The first type of DMEM (catalog no. 5671) contains 4.5 g/liter glucose and sodium bicarbonate, while the second type of DMEM (catalog no. 5546) contains 1 g/liter glucose and 0.11 g/liter pyruvic acid. Both media are devoid of Gln and Glu. These media were supplemented with histidine at 0.13 mg/ml, arginine at 0.72 mg/ml, leucine at 1 mg/ml, valine at 0.6 mg/ml, glucose at 0.15%, sodium acetate at 0.75 mg/ml, morpholinepropanesulfonic acid (MOPS) at 13 mg/ml, guanine at 0.05 mg/ml, xanthine at 0.05 mg/ml, FeSO_4_ at 0.1 mg/ml, ZnSO_4_ at 0.1 mg/ml, and adenine at 0.2 mg/ml. Unless stated otherwise, cells were washed and resuspended in KPM buffer (0.1 M K_2_HPO_4_ adjusted with H_3_PO_4_ acid to pH 6.5 and supplemented with 10 mM MgSO_4_) (46). Escherichia coli NEB-5α cells containing pGh9 derivatives were grown in HI medium containing 150 μg/ml Em at 30°C with aeration at 250 rpm.

### c-di-AMP extraction and quantification.

c-di-AMP from L. Lactis was extracted as previously described (25). c-di-AMP was detected and quantified by liquid chromatography-coupled tandem mass spectrometry (LCMS-8060; Shimadzu, Japan). Chromatographic separation was performed on an ultrahigh-pressure liquid chromatography (UHPLC) Nextera X2 instrument using a Shim-pack Velox SP-C_18_ column (1.8 μm, 2.1 by 150 mm; Shimadzu, Japan). Eluents A and B consisted of 0.05% (vol/vol) formic acid in water and acetonitrile (Merck), respectively. The sample volume was 10 μl with a flow rate of 0.3 ml min^−1^. Eluent A (95%) was used from 0 to 1 min, followed by a linear gradient from 95% to 50% eluent A until 10 min. The column was then washed with 90% eluent B for 3 min and then reequilibrated with 95% eluent A for 2 min prior to reinjection. The internal standard of azidothymidine (AZT) (Sigma) was used. c-di-AMP was detected with a triple-quadruple mass spectrometer equipped with an electrospray ionization source using multiple reaction monitoring transitions of *m/z* 657→124 in negative ionization mode. Data obtained were curated using LabSolutions Insight version 3.2 SP1 and LabSolutions Postrun/QuantBrowser version 5.95 (Shimadzu Corporation).

### Isolation of CEF-resistant suppressors and WGS.

The *cdaA* mutant strains *cdaA-1* and *cdaA-2* were streaked or spread either from mid-log-phase cultures, from broth cultures grown overnight, or directly from frozen glycerol stocks (40% glycerol) onto GM17 agar containing ≥0.08 μg/ml CEF (Merck) and incubated for 2 days at 30°C. Colonies were picked and restreaked on agar containing the same concentration of CEF, from where they were obtained to ensure purity. CEF resistance confirmation was carried out by serial dilution of mid-log-phase cultures onto GM17 agar with CEF.

CEF disk diffusion assays were carried out by mixing 5 μl of a mid-log-phase culture (optical density [OD] of ∼0.7) with 7 ml of 0.75% GM17 agar and pouring the culture onto a 15-ml 1.5% GM17 agar base. Following drying, a sterile 8-mm disk was placed on the top agar, and a 10-μl solution of CEF was added (0.15 μg). Following incubation overnight, zones of inhibition were observed. CEF-resistant suppressors were checked for *cdaA* back-mutations using PCR (Table S3), as described previously (22), before being analyzed by WGS. Genomic DNA extractions were performed as described previously (47). Sequencing was performed using the Illumina NovaSeq 6000 platform (Macrogen, South Korea). Single nucleotide polymorphisms (SNPs) were analyzed using Geneious Prime (Biomatters Ltd., New Zealand) (22, 25).

10.1128/mBio.00324-21.10 TABLE S3 Primers used in this study. Download Table S3, DOCX file, 0.01 MB. Copyright © 2021 Pham et al. 2021 Pham et al. https://creativecommons.org/licenses/by/4.0/ This content is distributed under the terms of the Creative Commons Attribution 4.0 International license.

### Genetic manipulation of strains.

Plasmids and primers used in this study are shown in Tables S2 and S3, respectively. Electroporation of L. lactis strains was done as previously described (48), with minor changes for some strains. Following electroporation of pGh9-*kupB* and pGh9-*glnP* into the *kupB-2* and *glnP-1* strains, respectively, cells were plated onto GM17 agar with 3 μg/ml Em supplemented with 0.1 M NaCl. The activities of promoters were determined using pTCV-lac (49). The WT promoter of *kupB* and the mutated variant from the *kupB-2* strain were amplified and cloned into pTCV-lac and assayed for activity in the L. lactis WT using a β-galactosidase assay.

### Isolation of l-5-*N*-hydroxyglutamine-resistant suppressors.

The *cdaA* mutant strain *cdaA-1* was streaked onto GM17 agar containing 1 mM the toxic glutamine analog l-5-*N*-hydroxyglutamine (Merck) and incubated for 2 days at 30°C. Colonies were picked and restreaked on agar containing the same concentration of the analog to ensure purity. Resistance confirmation was carried out by the serial dilution drop plate method as described above.

### Cell wall thickness analysis.

Cells grown to mid-log phase were fixed with 2.5% glutaraldehyde (ProSciTech) in phosphate-buffered saline (pH 7.4), and after washing in buffer, they were postfixed in 1% osmium tetroxide. They were then gradually dehydrated in ethanol (30 to 100%), infiltrated with a gradual increase in the concentration of Epon resin, and then polymerized for 2 days at 60°C. Ultrathin (60-nm) sections were collected onto 200-mesh copper grids and stained with uranyl acetate and lead citrate. Grids were examined using a Hitachi HT7700 electron microscope (Hitachi, Japan) operated at 80 kV. Images were acquired with a complementary metal oxide semiconductor (CMOS) camera (Advanced Microscopy Techniques), and peptidoglycan thickness was measured on micrographs.

### UDP-NAG quantitation.

Mid-log-phase (OD at 600 nm [OD_600_] of ∼0.6) cells were collected by centrifugation at 5,000 × *g* for 10 min at 4°C and washed 2 times with 1/10 KPM buffer. UDP-NAG was extracted and quantified as previously described (22).

### Peptidoglycan muropeptide and cross-linking analysis.

Peptidoglycan was extracted from exponential-phase cells (OD_600_ of ∼0.8) and then digested with mutanolysin as described previously (50). The resulting soluble muropeptides were reduced with sodium borohydride and separated by reverse-phase UHPLC (RP-UHPLC) with a 1290 chromatography system (Agilent Technologies) and a Zorbax Eclipse Plus C_18_ Rapid Resolution High Definition column (100 by 2.1 mm with a particle size of 1.8 μm; Agilent Technologies) at 50°C using ammonium phosphate buffer and a methanol linear gradient as described previously (51). Muropeptides were identified according to their retention times by comparison with an L. lactis muropeptide reference chromatogram (51). The different muropeptides were quantified by integration of the peak areas, and the percentage of each peak was calculated as the ratio of its area over the sum of all peak areas. The peptidoglycan cross-linking index was calculated according to methods described previously (52), as follows: (1/2 Σ dimers + 2/3 Σ trimers + 3/4 Σ tetramers)/Σ all muropeptides.

### Quantification of amino acid pools in growing L. lactis cells.

Cells were grown in 30 ml GM17 medium until an OD_600_ of ∼0.7 was reached, collected by centrifugation at 5,000 × *g* for 10 min at 4°C, and washed twice in 1/10 KPM buffer. After resuspension in 1.8 ml of 50% acetonitrile, cells were lysed using a Precellys 24 homogenizer (Bertin Technologies) with a 0.5-ml equivalent of 0.1-mm zirconia/silica beads (6,000 rpm for 30 s and repeated 3 times, with chilling on ice between repeats). Following centrifugation at 17,000 × *g* for 15 min at 4°C, the supernatant was mixed 1:1 with an internal standard of sarcosine and 2-aminobutanoic acid. Amino acids were derivatized and analyzed with an Agilent 1200-SL HPLC system with a fluorescence detector (FLD) (catalog no. G1321A; Agilent) as described previously (53).

### Extraction and quantification of intracellular Glu in the Gln uptake assay.

Cells were grown in 50 ml of CDM containing a low level of Gln (200 μM) until an OD_600_ of ∼0.4 was reached. Following centrifugation at 5,000 × *g* for 10 min at 25°C, cells were washed twice in KPM buffer and then resuspended in 3 ml of 1/10 KPM buffer. Uptake assays were performed using 0.5 ml of cells. Cells were energized with 20 mM glucose (final concentration) first before adding Gln (1 mM final concentration). The uptake assay mixture was incubated at 30°C for 5 min. Control reaction mixtures without glucose were included. For osmotic treatments, NaCl, KCl, and sucrose were added before Gln and incubated at 30°C for 20 min. After incubation, samples were centrifuged at 17,000 × *g* for 1 min at 4°C and washed twice with KPM buffer. Thereafter, Glu was extracted from cells using acetonitrile-methanol-H_2_O at a ratio of 2:2:1 using the same method as that described previously for c-di-AMP extraction (25). The supernatant (600 μl) was dried in an RVC 2-18 CDplus rotation vacuum concentrator (Christ). Glu and Gln were measured using the Glu assay kit (catalog no. MAK004-1KT; Merck) or the Gln and Glu determination kit (catalog no. GLN1-1KT; Merck), with minor adjustments to the protocols. For the Glu assay kit, the dried samples were resuspended in 50 μl of Glu assay buffer, vortexed well, and centrifuged at 16,000 × *g* for 5 min at 25°C. A portion (2 μl) was added into the kit master mix before incubation and reading of the OD_450_ using a NanoDrop One instrument (Thermo Fisher Scientific). For quantification, Glu standards were prepared at 1,000 to 31.25 μM in serial 2-fold dilutions.

### DNA/RNA release (lysis) assays during growth and under hypotonic conditions.

Strains were grown in HI broth until an OD_600_ of ∼0.2 was reached. HI broth was chosen instead of GM17 medium for this experiment as the latter produced fluorescent smears in the gels, making it less sensitive. The culture was split into 10-ml volumes, where CEF was added at different concentrations. Samples (100 μl) were collected every 2 h and then centrifuged at 17,000 × *g* for 3 min. The supernatant (20 μl) was taken and analyzed for the presence of genomic DNA and RNA by agarose gel electrophoresis using SYBR Safe stain (Invitrogen) and the 1-kb Plus DNA ladder (Thermo Fisher Scientific).

Lysis was also tested for strains suspended in hypotonic liquid. L. lactis strains were grown in GY broth with or without NaCl until an OD_600_ of ∼0.5 was reached. Cells (1.5 ml) were collected by centrifugation at 12,000 × *g* for 3 min, and the pellets were then washed with KPM followed by 1/10 KPM and centrifuged at 12,000 × *g* for 1 min. Cells were resuspended in 100 μl of MilliQ (Merck)-treated deionized water and centrifuged within 1 min at 17,000 × *g* for 3 min. The supernatant (20 μl) was taken and analyzed by agarose gel electrophoresis as described above.

The effect of Gln uptake on cell lysis in hypotonic liquid was also examined. WT and *cdaA-1* strains (15 ml) were grown to an OD_600_ of ∼0.6 in CDM broth containing a low level of Gln (100 μM), washed twice with KPM buffer before being resuspended in 1 ml of KPM buffer, and then divided into 2 500-μl aliquots for the sample and control. To obtain high internal Glu levels, 30 mM glucose and 10 mM Gln were added to cell suspensions and incubated at 30°C for 30 min. The control samples contained glucose but no Gln. After incubation, cells were centrifuged and washed with KPM buffer and then 1/10 KPM buffer at 12,000 × *g* for 1 min. The cell pellet was resuspended in 200 μl of MilliQ-treated deionized water and centrifuged within 1 min at 17,000 × *g* for 3 min. The supernatant (20 μl) was taken and analyzed by agarose gel electrophoresis as described above.
