# Supplementary material for: Cyclic di-AMP Oversight of Counter-Ion Osmolyte Pools Impacts Intrinsic Cefuroxime Resistance in Lactococcus lactis
Source: mBio. 2021 Apr 8;12(2):e00324-21. doi: 10.1128/mBio.00324-21 (PMC8092236; doi:10.1128/mBio.00324-21)
Supplement: TABLE S1 [file mBio.00324-21-st001.docx]

Table S1. Mutations identified in CEF suppressor mutants.

| **Parent** | **Suppressor** | **KupB**  K^+^  importer  (Llmg0588) | **GlnPQ** (glutamine ABC transporter)  (Llmg1942 & 1943) | **Other mutations** |
| --- | --- | --- | --- | --- |
| ***cdaA-2*** | *kupB-1* | T230I |  |  |
|  | *kupB-2* | G→T  27 bp upstream |  |  |
|  | *kupB-3* | V243G |  |  |
|  | *kupB-4* | T378I |  | Glycine betaine ABC transporter (BusAA; Llmg1048)  (insertion of *IS*905 38 bp upstream) |
|  | *kinF-1* |  |  | KinF (A219D) |
| ***cdaA-1*** | *glnP-1* |  | A603T (GlnP) |  |
|  | *glnP-2* |  | G52D (GlnP) |  |
|  | *glnP-3* |  | G569R (GlnP) |  |
|  | *glnP-4* |  | G52D (GlnP) | Ribosomal protein RpsU (D14N) |
|  | *glnP-5* |  | Q146E (GlnP) | Dipeptide transporter DtpP (Q146E) |
|  | *pstC-1* |  |  | Phosphate ABC transporter PstC (R202S) |
|  | *glnQ-1* |  | M194I (GlnQ) |  |
