# Supplementary figures and images for: Cyclic di-AMP Oversight of Counter-Ion Osmolyte Pools Impacts Intrinsic Cefuroxime Resistance in Lactococcus lactis
Source: mBio. 2021 Apr 8;12(2):e00324-21. doi: 10.1128/mBio.00324-21 (PMC8092236; doi:10.1128/mBio.00324-21)

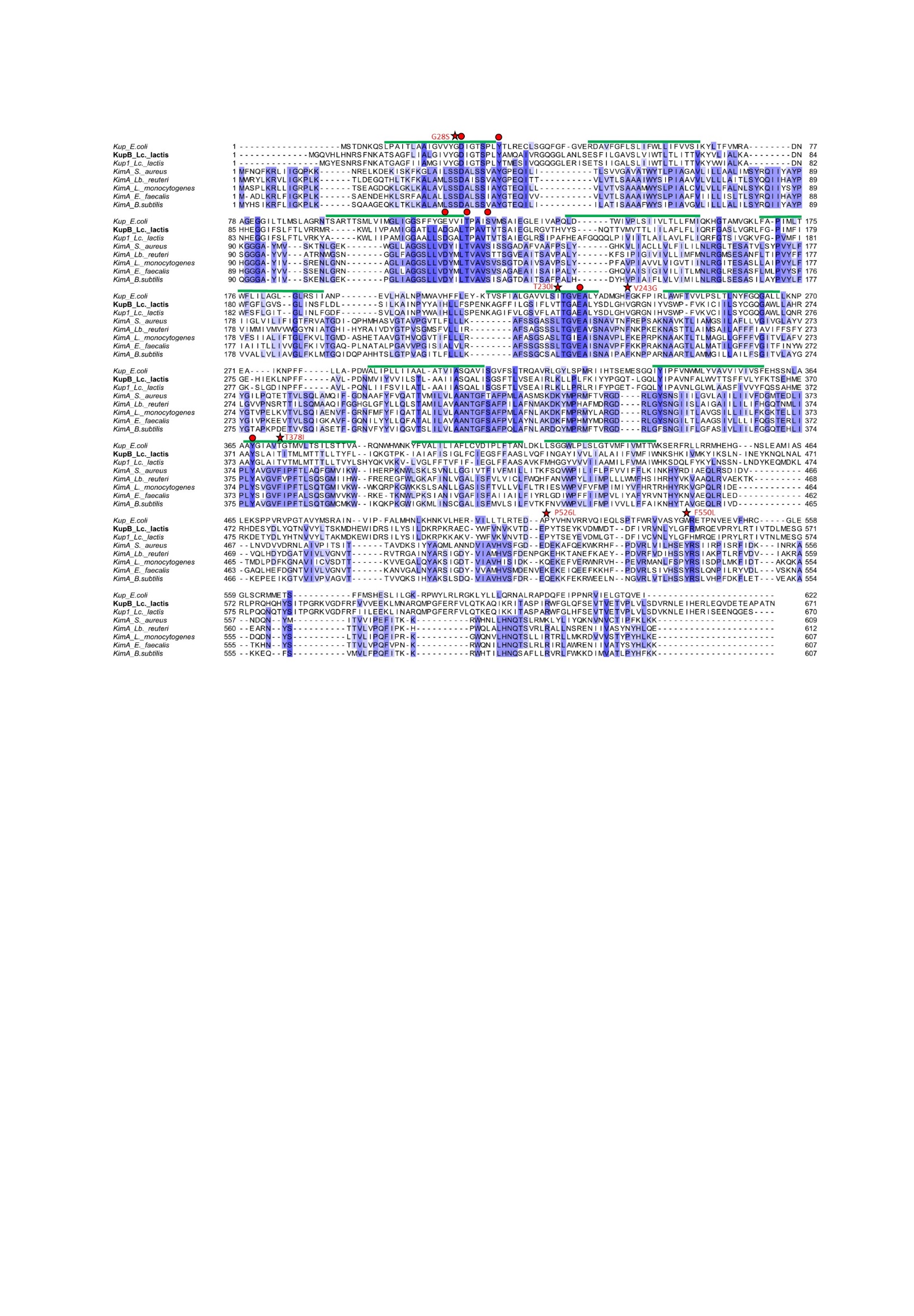

Supplement: FIG S2 [file mBio.00324-21-sf002.docx]
