# Supplementary material for: Cyclic di-AMP Oversight of Counter-Ion Osmolyte Pools Impacts Intrinsic Cefuroxime Resistance in Lactococcus lactis
Source: mBio. 2021 Apr 8;12(2):e00324-21. doi: 10.1128/mBio.00324-21 (PMC8092236; doi:10.1128/mBio.00324-21)
Supplement: TABLE S2 [file mBio.00324-21-st002.docx]

Table S2. Strains and plasmids used in this study.

| Strains | Phenotype | Antibiotic resistance | Source |
| --- | --- | --- | --- |
| WT | *L. lactis* subsp. *cremoris* MG1363, laboratory strain, plasmid cured |  |  |
| *gdpP-1* | This whole genome sequenced strain derived from WT contains a *llmg_1768* deletion and spontaneous *gdpP^A573D^*  and *rdrA* mutations. Contains high c-di-AMP and is osmosensitive. Previously called OS1 (1). |  | (2) |
| *gdpP-2* | This whole genome sequenced strain derived from WT has one spontaneous mutation (*gdpP^K122Stop^*). Contains high c-di-AMP and is osmosensitive. Previously called Δ*gdpP* (3) and OS2 (1). |  | (1, 2) |
| *cdaA-1* | Salt resistant suppressor of *gdpP-1*. Genome sequenced previously. Compared to *gdpP-1* it contains a mutation in *cdaA* (1-bp deletion in codon 11) and G→T mutation 142-bp upstream of *busAA*. This *busAA* mutation is located in the promoter and turns transcription off. Contains very low c-di-AMP. Previously called OR1 (1). |  | (1) |
| *cdaA-2* | Salt resistant suppressor mutant of *gdpP-2*. Genome sequenced in this study. Compared to *gdpP-2* it contains one mutation (*cdaA^A186V^*). Contains reduced c-di-AMP compared to *gdpP-2*. |  | This study |
| *kupB-1*, *kupB-2*, *kupB-3*, *kupB-4* and *kinF-1* | CEF resistant suppressor of *cdaA-2*. All were genome sequenced in this study. |  | This study |
| *glnP-1*, *glnP-2*, *glnP-3*, *glnP-4*, *glnP-5*, *ptsC1* and *glnQ-1* | CEF resistant suppressor of *cdaA-1*. All were genome sequenced in this study. |  | This study |
| *glnP-6* | Toxic glutamine analog (L-5-N-hydroxyglutamine) resistant suppressor from *cdaA-1*. Contains *glnP* mutation (C→T mutation resulting in a change from Q442 to a stop codon (CAA to TAA). |  | This study |
| *glnP-7* | Toxic glutamine analog (L-5-N-hydroxyglutamine) resistant suppressor from *cdaA-1***.** Contains *glnP* mutation (C→T mutation resulting in a change from Q44 to a stop codon (CAA to TAA). |  | This study |
| WT-pGh9 | WT containing pGh9 | Em^r^ | (3) |
| *gdpP-2*-pGh9 | *gdpP-2* containing pGh9 | Em^r^ | (3) |
| *cdaA-2*-pGh9 | *cdaA-2* containing pGh9 | Em^r^ | This study |
| *kupB-2*-pGh9 | *kupB-2* containing pGh9 | Em^r^ | This study |
| *kupB-2*-pGh9-*kupB* | *kupB-2* overexpressing *kupB* from pGh9-*kupB* | Em^r^ | This study |
| *glnP-1*-pGh9-*glnP* | *glnP-1* overexpressing *glnP* from pGh9-*glnP* | Em^r^ | This study |
| *glnP-1*-pGh9-*glnP* (cured) | *glnP-1*-pGh9-*glnP* was cured of pGh9-*glnP*. Contains no plasmid and is equivalent to *glnP-1* (Em^s^). |  | This study |
| WT pTCV-lac-*P_busAA_-lacZ* | WT containing pTCV-lac-*P_busAA_-lacZ* | Kan^r^, Em^r^ | This study |
| WT pTCV-lac-*P_busAA_^-142G→T^-lacZ* | WT containing pTCV-lac-*P_busAA_^-142G→T^-lacZ* | Kan^r^, Em^r^ | This study |
| WT pTCV-lac-P*_kupB_*-*lacZ* | WT containing pTCV-lac-P*_kupB_*-*lacZ* | Kan^r^, Em^r^ | This study |
| WT pTCV-lac-*P_kupB_^-27G→T^-lacZ* | WT containing pTCV-lac-*P_kupB_^-27G→T^-lacZ* | Kan^r^, Em^r^ | This study |
| *gdpP-2*-*kupB ^A618V^* | Salt resistant suppressor mutant of *gdpP-2*. Genome sequenced previously (previously called Δ*gdpP-kupB^A618V^*). Compared to *gdpP-2* it contains one mutation (*kupB ^A618V^*). Contains higher K^+^ level and c-di-AMP compared to *gdpP-2*. |  | (3) |
| *gdpP-2* pGh9-*kupB^A618V^* | *gdpP-2* overexpressing *kupB^A618V^* from pGh9-*kupB^A618V^* | Em^r^ | (3) |
| *E. coli* NEB-5α | Routine DNA cloning strain. |  | New England Biolabs |
| pGh9 | Previously called pPNG904. A pGhost9::IS*S1* derivative containing a Cm^r^ gene inserted into the EcoRI and SalI sites, replacing IS*S1*. Replicates at 30°C in *L. lactis* and *E. coli*. | Em^r^ | (4) |
| pGh9-*kupB* | pGh9 containing entire WT *kupB* replacing the Cm^r^ gene | Em^r^ | (3) |
| pGh9-*kupB ^A618V^* | pGh9 containing entire *kupB^A618V^* replacing the Cm^r^ gene | Em^r^ | (3) |
| pGh9-*glnP* | pGh9 containing entire WT *glnP* replacing the Cm^r^ gene | Em^r^ | This study |
| pTCV-lac | Shuttle vector with a promoter-less *lacZ* gene. | Kan^r^, Em^r^ | (5) |
| pTCV-lac-*P_busAA_-lacZ* | pTCV-lac with the WT *busAA* promoter fused to the *lacZ* reporter gene | Kan^r^, Em^r^ | (3) |
| pTCV-lac-*P_busAA_^-142G→T^-lacZ* | pTCV-lac with the *busAA* promoter from *cdaA-1* (contains a G→T mutation 142-bp upstream of *busAA*) fused to the *lacZ* reporter gene | Kan^r^, Em^r^ | This study |
| pTCV-lac-P*_kupB_*_*lacZ* | pTCV-lac with the WT *kupB* promoter fused to the *lacZ* reporter gene | Kan^r^, Em^r^ | This study |
| pTCV-lac-*P_kupB_^-27G→T^-lacZ* | pTCV-lac with the *kupB* promoter from *kupB-2* (contains a G→T mutation 27-bp upstream of *kupB*) fused to the *lacZ* reporter gene | Kan^r^, Em^r^ | This study |

1. Zhu Y, Pham TH, Nhiep THN, Vu NMT, Marcellin E, Chakrabortti A, Wang Y, Waanders J, Lo R, Huston WM, Bansal N, Nielsen LK, Liang Z-X, Turner MS. 2016. Cyclic-di-AMP synthesis by the diadenylate cyclase CdaA is modulated by the peptidoglycan biosynthesis enzyme GlmM in *Lactococcus lactis.* Mol Micro 99:1015–1027.

2. Smith WM, Pham TH, Lei L, Dou J, Soomro AH, Beatson SA, Dykes GA, Turner MS. 2012. Heat resistance and salt hypersensitivity in *Lactococcus lactis* due to spontaneous mutation of *llmg_1816* (*gdpP*) induced by high-temperature growth. Appl Environ Microbiol 78:7753-9.

3. Pham HT, Nhiep NTH, Vu TNM, Huynh TN, Zhu Y, Huynh ALD, Chakrabortti A, Marcellin E, Lo R, Howard CB, Bansal N, Woodward JJ, Liang ZX, Turner MS. 2018. Enhanced uptake of potassium or glycine betaine or export of cyclic-di-AMP restores osmoresistance in a high cyclic-di-AMP *Lactococcus lactis* mutant. PLoS Genet 14:e1007574.

4. Lo R, Turner MS, Barry DG, Sreekumar R, Walsh TP, Giffard PM. 2009. Cystathionine gamma-lyase is a component of cystine-mediated oxidative defense in *Lactobacillus reuteri* BR11. J Bacteriol 191:1827-37.

5. Poyart C, Trieu-Cuot P. 1997. A broad-host-range mobilizable shuttle vector for the construction of transcriptional fusions to beta-galactosidase in Gram-positive bacteria. Fems Microbiology Letters 156:193-8.
