# Supplementary material for: Cyclic di-AMP Oversight of Counter-Ion Osmolyte Pools Impacts Intrinsic Cefuroxime Resistance in Lactococcus lactis
Source: mBio. 2021 Apr 8;12(2):e00324-21. doi: 10.1128/mBio.00324-21 (PMC8092236; doi:10.1128/mBio.00324-21)
Supplement: TABLE S3 [file mBio.00324-21-st003.docx]

Table S3. Primers used in this study.

| Primer name | Sequence | Target |
| --- | --- | --- |
| *glnP*_F_OE | ATT**CTGCAG**GTCAGAAAACCTAACATCAAC | For cloning full length *glnP* into pGh9 and for checking the sequence of *glnP* |
| *glnP*_R_OE | CTC**CTCGAG**GTATTGATTCCCATGTTAG | For cloning full length *glnP* into pGh9 and for checking the sequence of *glnP* |
| *glnP*_mid | GCTACAAACTTCCCTTCTATC | For checking the sequence of *glnP* |
| *glnQ*_F | GTTCTTCTCTTCTTGATGTGG | For checking the sequence of *glnQ* |
| *glnQ*_R | GCTTATGGAATCTTACGTGCC | For checking the sequence of *glnQ* |
| *kupB*-P_F | AAT**GAATTC**ATTCACGAGCGAATTTCTAAGG | For cloning the *kupB* promoter into pTCV-lac |
| *kupB*-P_R | TTT**GGATCC**GCTCGAAAACGACTTGCACGC | For cloning the *kupB* promoter into pTCV-lac |
| *busAA-*P_F | CTT**GAATTC**CGTAGGAGCTTCTGATACAGG | For cloning the *busAA* promoter into pTCV-lac |
| *busAA*-P_R | AAA**GGATCC**CATTCTATTACTCATGAGCCG | For cloning the *busAA* promoter into pTCV-lac |
