# Supplementary material for: Cyclic di-AMP Oversight of Counter-Ion Osmolyte Pools Impacts Intrinsic Cefuroxime Resistance in Lactococcus lactis
Source: mBio. 2021 Apr 8;12(2):e00324-21. doi: 10.1128/mBio.00324-21 (PMC8092236; doi:10.1128/mBio.00324-21)
Supplement: Supplementary file 5 [file mBio.00324-21-sf001.docx]

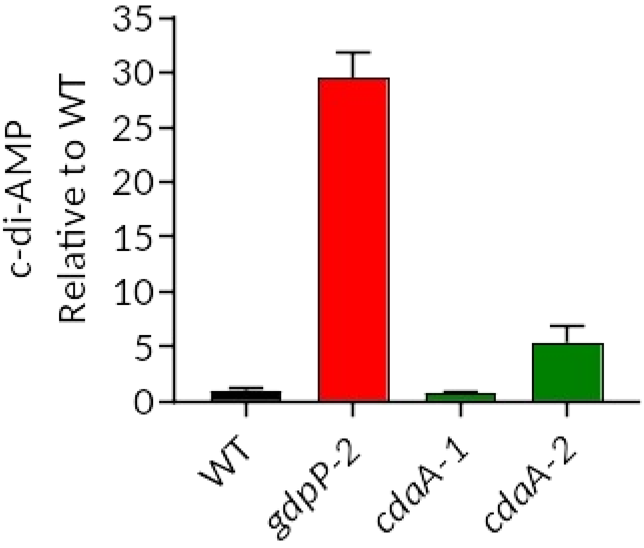
c-di-AMP
Relative to WT
35
30
25
20
15
10
5
0
WT
gdpP-2
cdaA-1
cdaA-2
