# Supplementary material for: Cyclic di-AMP Oversight of Counter-Ion Osmolyte Pools Impacts Intrinsic Cefuroxime Resistance in Lactococcus lactis
Source: mBio. 2021 Apr 8;12(2):e00324-21. doi: 10.1128/mBio.00324-21 (PMC8092236; doi:10.1128/mBio.00324-21)
Supplement: Supplementary file 6 [file mBio.00324-21-sf003.docx]

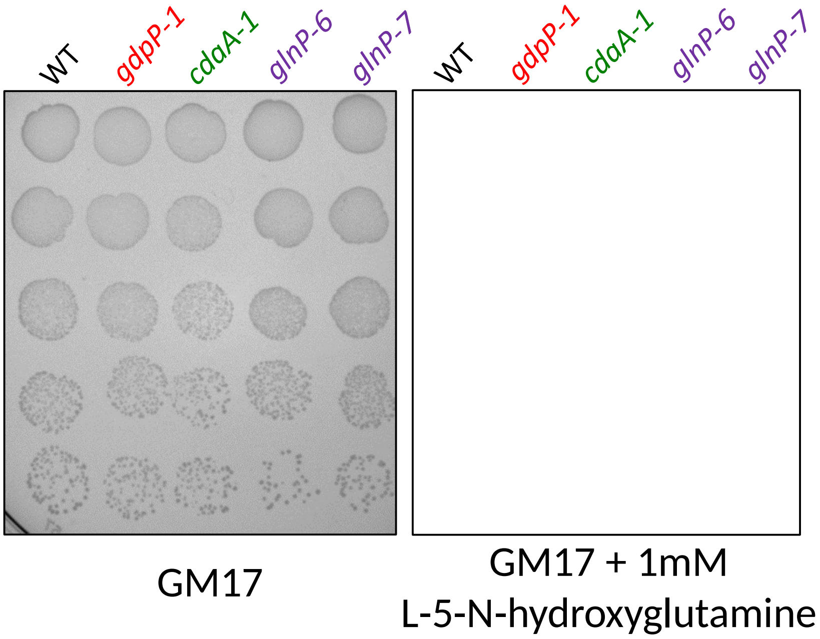

WT
gdpP-1
cdaA-1
glnP-6
glnP-7
WT
gdpP-1
cdaA-1
glnP-6
glnP-7
GM17
GM17 + 1mM
L-5-N-hydroxyglutamine
