# Supplementary material for: Cyclic di-AMP Oversight of Counter-Ion Osmolyte Pools Impacts Intrinsic Cefuroxime Resistance in Lactococcus lactis
Source: mBio. 2021 Apr 8;12(2):e00324-21. doi: 10.1128/mBio.00324-21 (PMC8092236; doi:10.1128/mBio.00324-21)
Supplement: Supplementary file 7 [file mBio.00324-21-sf004.docx]

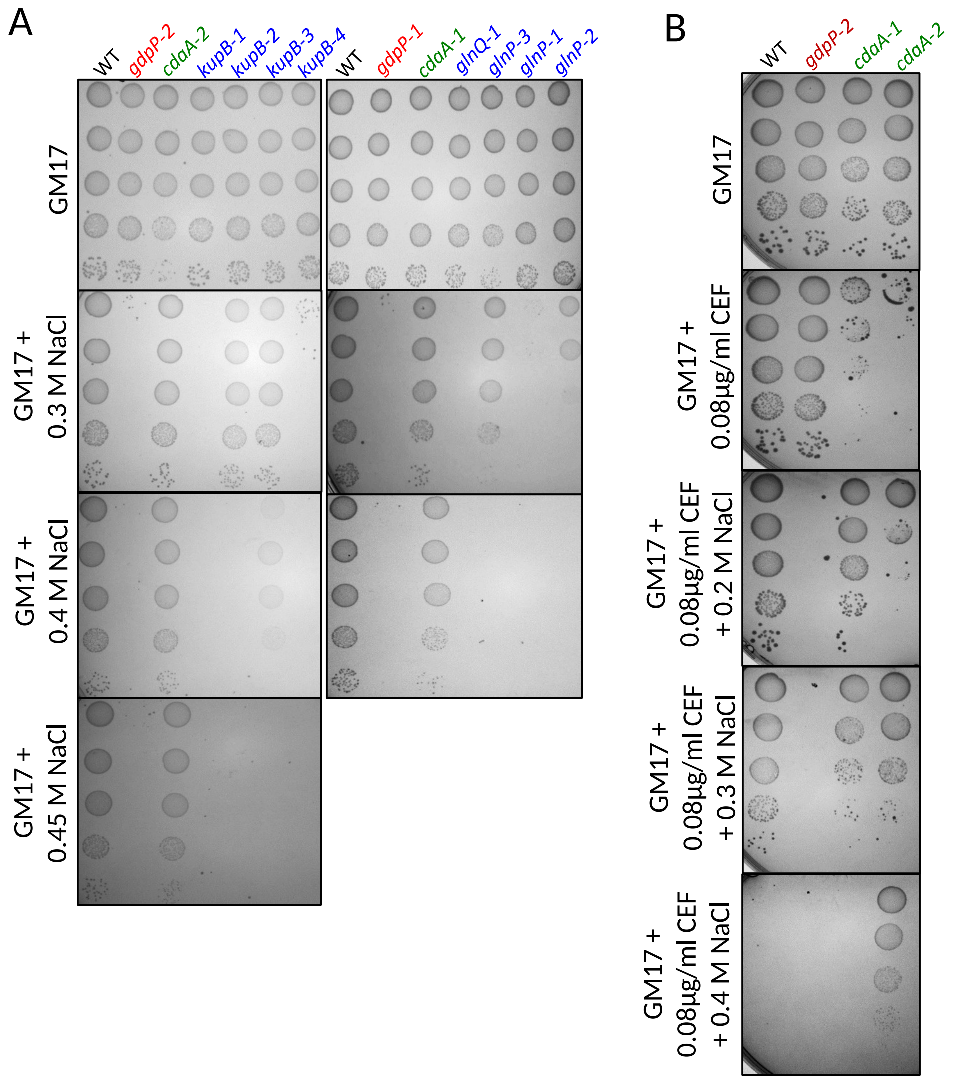
A
WT
gdpP-2
cdaA-2
kupB-1
kupB-2
kupB-3
kupB-4
WT
gdpP-1
cdaA-1
glnQ-1
glnP-3
glnP-1
glnP-2
GM17
GM17 + 0.3 M NaCl
GM17 + 0.4 M NaCl
GM17 + 0.45 M NaCl
B
WT
gdpP-2
cdaA-1
cdaA-2
GM17
GM17 + 0.08µg/ml CEF
GM17 + 0.08µg/ml CEF + 0.2 M NaCl
GM17 + 0.08µg/ml CEF + 0.3 M NaCl
GM17 + 0.08µg/ml CEF + 0.4 M NaCl
