# Supplementary material for: Cyclic di-AMP Oversight of Counter-Ion Osmolyte Pools Impacts Intrinsic Cefuroxime Resistance in Lactococcus lactis
Source: mBio. 2021 Apr 8;12(2):e00324-21. doi: 10.1128/mBio.00324-21 (PMC8092236; doi:10.1128/mBio.00324-21)
Supplement: Supplementary file 8 [file mBio.00324-21-sf005.docx]

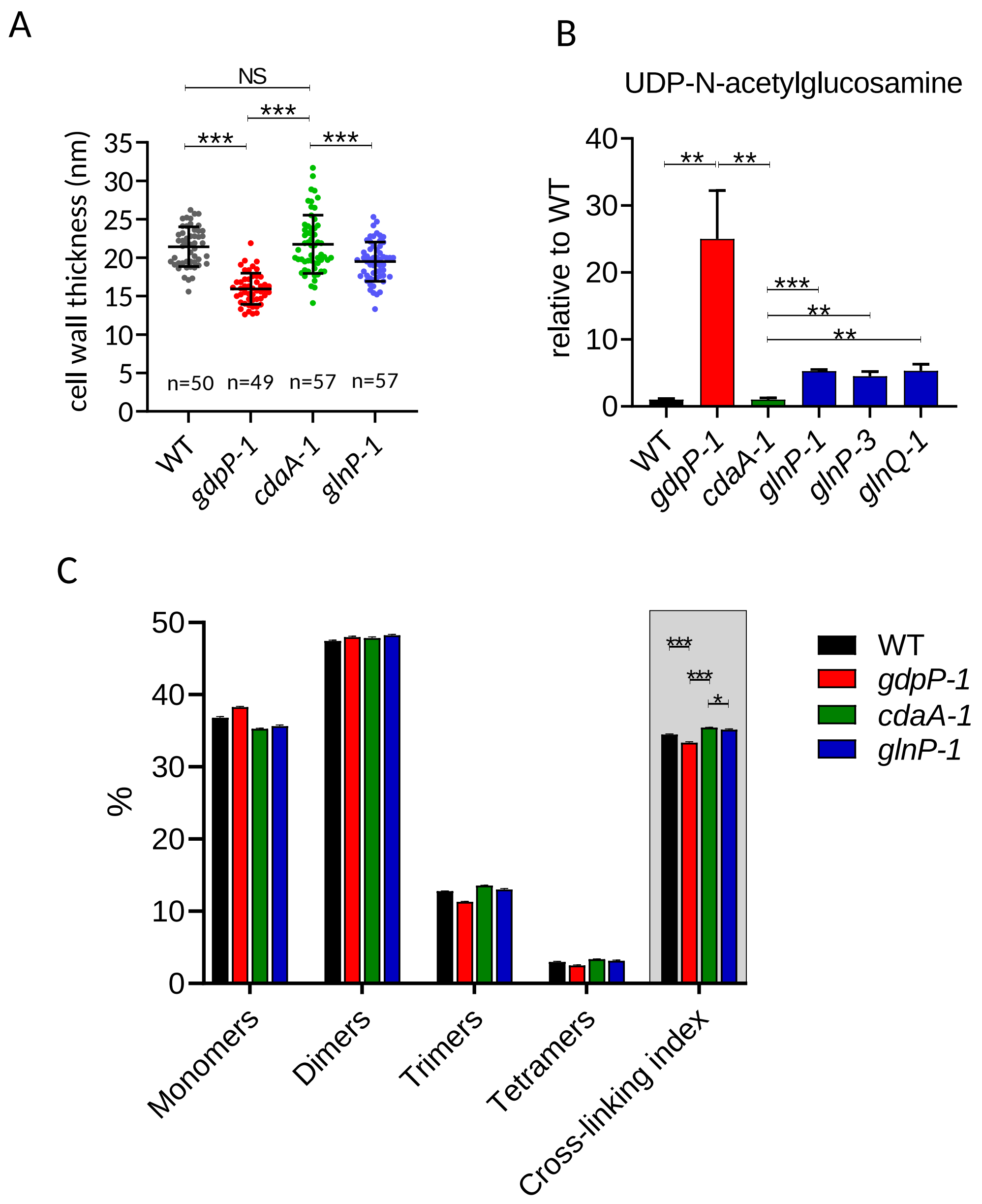
A
NS
***
***
***
cell wall thickness (nm)
35
30
25
20
15
10
5
0
n=50 n=49 n=57 n=57
WT
gdpP-1
cdaA-1
glnP-1
B
UDP-N-acetylglucosamine
relative to WT
40
30
20
10
0
**
**
***
**
**
WT
gdpP-1
cdaA-1
glnP-1
glnP-3
glnQ-1
C
%
50
40
30
20
10
0
***
***
*
Monomers
Dimers
Trimers
Tetramers
Cross-linking index
WT
gdpP-1
cdaA-1
glnP-1
