# Supplementary material for: Cyclic di-AMP Oversight of Counter-Ion Osmolyte Pools Impacts Intrinsic Cefuroxime Resistance in Lactococcus lactis
Source: mBio. 2021 Apr 8;12(2):e00324-21. doi: 10.1128/mBio.00324-21 (PMC8092236; doi:10.1128/mBio.00324-21)
Supplement: Supplementary file 9 [file mBio.00324-21-sf006.docx]

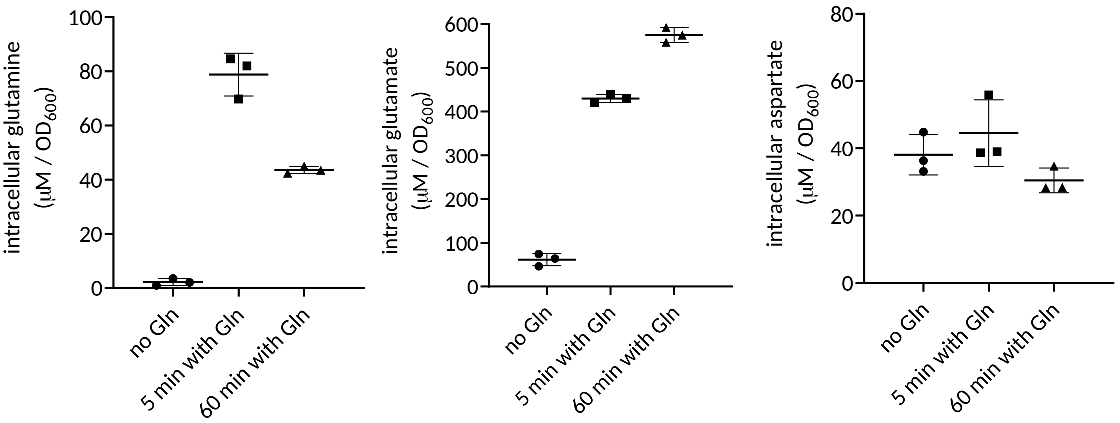

intracellular glutamine
(μM / OD600)
100
80
60
40
20
0
no Gln
5 min with Gln
60 min with Gln
intracellular glutamate
(μM / OD600)
600
500
400
300
200
100
0
no Gln
5 min with Gln
60 min with Gln
intracellular aspartate
(μM / OD600)
80
60
40
20
0
no Gln
5 min with Gln
60 min with Gln
