# Supplementary material for: Cyclic di-AMP Oversight of Counter-Ion Osmolyte Pools Impacts Intrinsic Cefuroxime Resistance in Lactococcus lactis
Source: mBio. 2021 Apr 8;12(2):e00324-21. doi: 10.1128/mBio.00324-21 (PMC8092236; doi:10.1128/mBio.00324-21)
Supplement: Supplementary file 10 [file mBio.00324-21-sf007.docx]

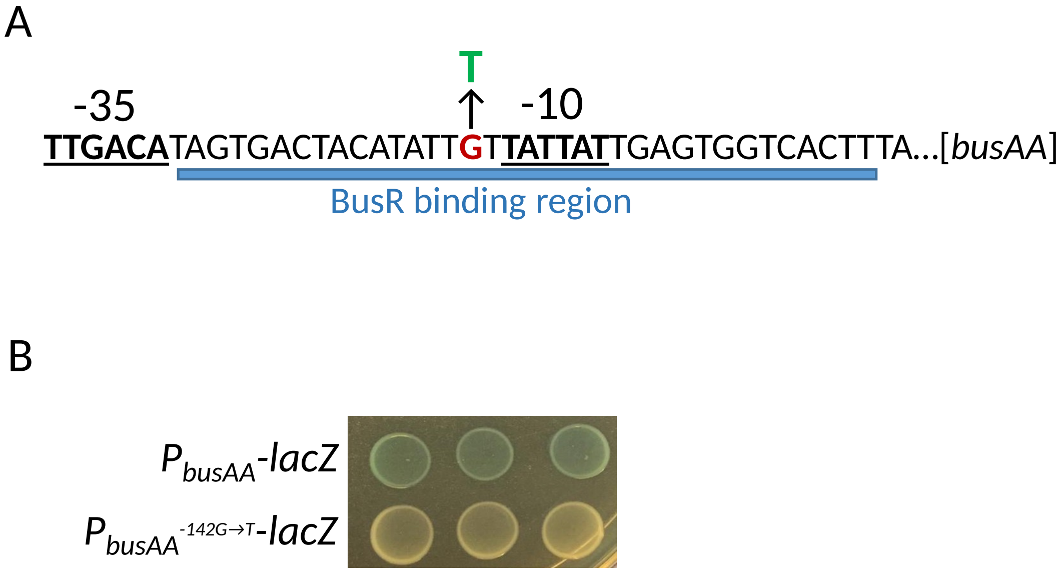
A
T
-35
-10
TTGACATAGTGACTACATATTGTTATTATTGAGTGGTCACTTTA…[busAA]
BusR binding region
B
P busAA-lacZ
P busAA -142G→T-lacZ
